# Physiologically based pharmacokinetic modelling to predict artemether and lumefantrine exposure in neonates weighing less than 5 kg treated with artemether–lumefantrine to supplement the clinical data from the CALINA study

**DOI:** 10.1186/s41182-025-00790-w

**Published:** 2025-08-25

**Authors:** Helen Gu, Nada Abla, Vinay Kumar Venishetty, Birgit Schoeberl, Julia Zack, Heidi J. Einolf

**Affiliations:** 1 Novartis Pharmaceuticals Corporation, One Health Plaza, East Hanover, NJ 07936-1080 USA; 2 MMV Medicines for Malaria Venture, Geneva, Switzerland; 3 Novartis Healthcare Private Limited, Biomedical Research, Hyderabad, India; 4 Novartis Institutes for Biomedical Research, Cambridge, MA USA

**Keywords:** Physiologically based pharmacokinetic modelling, Cytochrome P450 CYP3A4 ontogeny, Falciparum malaria, Neonatal malaria, Artemether–lumefantrine, Simcyp

## Abstract

**Background:**

Evidence-based recommendations for malaria treatment in patients weighing < 5 kg are lacking as a consequence of differences in pharmacokinetics due to age and/or body weight (BW), and recruitment challenges in conducting trials in this population. A physiologically based pharmacokinetic (PBPK) model was developed and validated to predict artemether and lumefantrine concentrations in patients < 5 kg BW aged 1–28 days. The model predictions supplemented data from a trial (CALINA; NCT04300309) with an optimized dose of artemether–lumefantrine (5 mg artemether: 60 mg lumefantrine) in patients < 5 kg with *Plasmodium falciparum* malaria.

**Methods:**

PBPK models of artemether and lumefantrine were developed using Simcyp (Version 22) and validation was performed using historical data from adults and paediatric patients. To compare model-predicted and observed values, populations were matched to clinical trial populations (ranging from adults to infants) for patient numbers and demographics. The models were applied to predict artemether maximal concentration (C_max_) and lumefantrine C_max_ and Day 7 concentration (C_168h_) in neonates of < 5 kg BW aged 1–28 days, and for subgroups aged 1–7, 8–14, and 15–28 days.

**Results:**

Validated models for artemether and lumefantrine were used to predict plasma concentrations in neonates and young infants with BW < 5 kg after 3-day administration of 5 mg artemether and 60 mg lumefantrine twice daily with high confidence. The PBPK model using Upreti hepatic cytochrome P450 (CYP)3A4 ontogeny predicted observed artemether and lumefantrine exposure in infants and neonates better than Salem ontogeny. The predicted variability in neonates was comparable to or larger than the variability of observed concentrations in infants and older neonates in the CALINA study.

**Conclusions:**

Based on the success of the PBPK models for artemether and lumefantrine in predicting drug concentrations in adults and children, including neonates, modelling and simulation results can be used with confidence to supplement the limited available data for neonates (1–28 days old) < 5 kg BW obtained from the CALINA study for this rarer and more difficult to recruit patient population.

**Supplementary Information:**

The online version contains supplementary material available at 10.1186/s41182-025-00790-w.

## Background

Malaria in neonates and infants < 12 months of age remains relatively poorly understood. In neonates, malaria can occur as a congenital infection transferred from an infected mother or may be acquired from a mosquito bite; cases in patients ≤ 7 days of age are considered to be congenital [1]. In malaria-endemic areas, infants < 3 months of age or approximately 5 kg of BW are often perceived to be protected from malaria due to the presence of maternal antibodies, which may result in misdiagnosis and mismanagement of the disease in this age group [1, 2]. The overall incidence of malaria in this age group is not well documented. For congenital malaria, reported global rates range from 0.1–0.6% to 3.7 to 22% [3–5]. While malaria infection can often be asymptomatic in young infants, it may cause anaemia [6] and poorer cognitive development [7].

Current treatment recommendations for this age group are limited. WHO guidelines for patients < 5 kg BW recommend treatment with artemisinin-based combination therapy (ACT) at the same mg/kg BW target dose as for children weighing ≥ 5 kg. However, this recommendation is not evidence based [8]. At the time of writing, there are no approved malaria treatments for patients with a BW < 5 kg, with the exception of artesunate/amodiaquine which is approved for patients > 4.5 kg. Moreover, most clinical studies of antimalarial treatment exclude infants of < 6 months of age or < 5 kg BW. National treatment guidelines vary widely [9]. Dose adjustment based on age or weight alone is not ideal for neonates and infants due to lower immunity [10] and differing PK profiles, such as higher clearance (CL) rates and maturational changes in metabolizing enzyme ontogeny may impact on the PK of these compounds. Dosage and treatment duration for patients < 6 months of age or weighing < 5 kg therefore cannot be extrapolated accurately from older children or adults based on per kg BW basis [10]. The absence of age- and weight-appropriate formulations of ACTs may lead to inappropriate dosing [10–12], thereby potentially posing a safety risk. There is a clear unmet need for age-appropriate dosing recommendations and suitable formulations for antimalarial treatment in infants and neonates with acute uncomplicated *P. falciparum* malaria and a BW < 5 kg.

The currently available artemether–lumefantrine dispersible tablet (20 mg artemether plus 120 mg lumefantrine; Coartem^®^ Dispersible, Novartis, Basel) was developed for paediatric patients with a BW of 5 to 35 kg and demonstrated a similar efficacy, safety and pharmacokinetics to crushed tablets [13, 14]. A study in patients > 28 days old and < 5 kg BW with uncomplicated falciparum malaria treated with artemether–lumefantrine dispersible tablets at the same dose as used for ≥ 5 kg paediatric patients reported 2- to 3-fold higher artemether and dihydroartemisinin (DHA, the active and major metabolite of artemether) systemic exposures than those seen in infants and children ≥ 5 kg [15]. Halving the dose was not considered to reduce the risk of artemether and DHA toxicity (given that C_max_ values would remain elevated) and the decreased lumefantrine exposure was highlighted as a risk for treatment failure [15]. A paediatric formulation with optimized dose for patients < 5 kg, with each dispersible tablet containing 2.5 mg artemether and 30 mg lumefantrine, was therefore developed based on modelling of drug exposures. A Phase II/III study (CALINA; COA566B2307; NCT04300309) was performed to assess the pharmacokinetics, efficacy, and safety of this optimized dose in infants and neonates of < 5 kg BW. The main study results are described elsewhere [9]. The CALINA study aimed to provide exposure similar to that achieved with the approved adult or currently available paediatric dosing regimen(s) to allow extrapolation of safety and efficacy from historical studies, an approach that is well-accepted for paediatric drug development. However, due to challenges in recruitment, the number of neonates enrolled was less than originally planned. Hence, PBPK models for artemether and lumefantrine were developed and validated to predict the exposures of artemether and lumefantrine in order to supplement the limited data available from the clinical study.

In this publication, we describe an update to the previous artemether PBPK model [16] and development of a lumefantrine PBPK model, validation and application of these models to predict the artemether and lumefantrine exposures. The primary endpoint in the CALINA study [9] was artemether C_max_, which is also associated with early parasite clearance, and higher concentrations may cause potential neurotoxicity. Secondary endpoints in the study were lumefantrine C_max_ and lumefantrine C_168h_; the former was assessed due to potential effects on safety, and the latter is an accepted marker for the 28-day cure rate. These parameters were of primary interest to predict in the PBPK model and are discussed in detail. The PBPK model was also developed to predict DHA concentrations; Additional File 1 summarizes the methodology and results for DHA.

## Methods

**Fig. 1:**
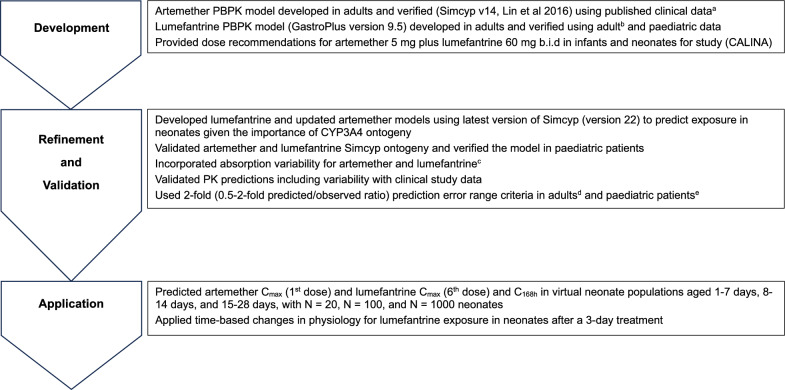
Artemether–lumefantrine overall PBPK modelling strategy. ^a^[13, 15], ^b^[17], ^c^[18, 19], ^d^[20, 21], ^e^[22, 23]

The overall PBPK modelling strategy, including model development, validation, and application, is illustrated in Fig. 1. Dose recommendations for the CALINA study (CCOA566B2307, NCT04300309 [9]) were based on PBPK models for artemether and lumefantrine in adults and verified using published data. Clinical data from the CALINA study were then used, together with data from other studies, to update, refine and validate models, which were then applied to predict drug exposures in neonatal malaria patients to support dosage recommendations.

The Simcyp Population-based Simulator (Version 22, Certara, L.P., Sheffield, UK) for both adults and paediatrics was used in this modelling analysis. For model validation of the PK predictions, the simulated and observed artemether and lumefantrine concentration–time course, C_max_ and/or AUC with variability were compared to the observed data from adults (Study CCOA566B2104 [24]) and paediatric patients (CCOA566B2303 [13]) and in children ≥ 5 kg BW; CCOA566B2306 (EudraCT 2011–005852-33 [15]) in infants < 5 kg and > 28 days of age, and CCOA566B2307 (CALINA, NCT04300309 [9]) in infants < 5 kg and > 28 days and older neonates (< 5 kg and < 28 days)), using the exact demographic variables including age, weight, gender, and height. Study CCOA566B2104 [24] was the only study providing data from adults to be included, as this is the only study in adults to utilize crushed tablets and dispersible tablets, which were used in the paediatric studies.

### Artemether model

The PBPK model using Simcyp Version 14, previously developed and validated to predict the PK of artemether in paediatrics [16], was updated to Simcyp Version 22. The model input parameters are summarized in Table 1.

**Table 1:** PBPK model input parameters for artemether

Parameter (unit)	Value	Source reference
*Physicochemical and plasma binding*		
Molecular weight (g/mol)	298.38	
logP	3.28	[16]
Compound type	Neutral	
B/P	0.8	[16, 25]
fu_p_	0.046	[16, 26]
*Absorption*		
Model used	First order absorption	
f_a_ (CV 91%)	1	[16] with variability inputs from [18], vide infra
*k*_a_ (1/h, CV 63%)	0.37	
P_eff,man_ (10^–4^ cm/s)	4.27	[16]
fu_gut_	0.046	
Q_gut_	14.4	Simcyp predicted, Version 22
*Distribution (full PBPK)*		
V_ss_ (L/kg)	5.95	Full PBPK “method 1” predicted with K_p_ scalar = 2
*Elimination*		
CYP3A4 CL_int_ (µL/min/pmol of isoform)	2.27	Retrograde model was used with CL_po_ = 102 L/h [27] and ~ 60% contribution of metabolized by CYP3A4 (fm_CYP3A4_)—calculated from the AUC ratio of ~ 2.4–2.5 with ketoconazole [28] and remaining fm_CYP2B6_ of ~ 40% [28, 29]
CYP2B6 CL_int_ (µL/min/pmol of isoform)	12.2	
CL_r_ (L/h)	0	[30]

Simcyp default CV% was used for all parameters, unless noted otherwise

### Lumefantrine model

A PBPK model of lumefantrine was initially developed and validated to predict the PK of lumefantrine in adults (CCOA566B2104). The input parameters for lumefantrine are summarized in Table 2.

**Table 2:** PBPK model input parameters for lumefantrine

Parameter (unit)	Value	Source
*Physicochemical and plasma binding*		
Molecular weight (g/mol)	528.94	
logP	8.62	ADMEt predicted
Compound type	Monoprotic base	
pK_a_1	9.35	[31]
B/P	0.8	[25]
fu_p_	0.003	[26]
*Absorption (first order absorption model)*		
f_a_ (CV 45%)	0.05 (fasted) 0.1 (milk intake in infants and neonates) CALINA(60 mg), B2303, BW of 5–15 kg and B2306 (120 mg) 0.2 (light meal in children) B2303, BW of 15–25 kg (240 mg) and BW of 25–35 kg (360 mg) 1 (fed) B2104 in adults	CV% input from [19], vide infra [17] [32] [33]
*k*_a_ (1/h, CV 0%)	0.13	
T_lag_ time (h)	2	[32]
P_eff,man_ (10^–4^ cm/s)	8.276	PSA 23 Å, HBD 1
fu_gut_	1	Assumption
Q_gut_	17.67	Simcyp predicted
*Distribution (minimal PBPK)*		
Q (L/h)	0.22	Initial estimated based on [34] and optimized to fit adult data CCOA566B2104 (dispersible tablet)
V_sac_ (L/kg)	1.38	
V_ss_ (L/kg)	1.60	
*Elimination*		
CL_int_ (µL/min/pmol of isoform) CYP3A4	0.314 (× 0.7 fu_mic_)	Retrograde model was used and derived from CL_iv_ of 0.79 L/h with fu_mic_ of 0.7 to achieve a contribution of 51% of CYP3A4 and 49% of other enzymes [28, 35]. The fmCYP3A4 was also confirmed by internal drug interaction study with itraconazole
CL_int_ (µL/min/mg protein) HLM	38.08 (× 0.7 fu_mic_)	
CL_r_ (L/h)	0	CCOA566B2106
*Interaction*		
CYP2D6 competitive inhibition *K*_i_,_u_ (µM)	0.00138	

Simcyp default CV% was used for all parameters, unless noted otherwise

*PSA* polar surface area, *HBD* hydrogen bond donor, *HLM* human liver microsomes

### Refinement and validation

Both PBPK models were validated to simulate appropriately artemether and lumefantrine plasma concentrations based on the observed data from the studies shown in Table 3.

**Table 3:** Trials used for PBPK model validation in the studies conducted using dispersible tablets

Study	Key study objective/number of patients	Dose regimen	Measured PK parameters/sampling schedule	Use in PBPK model
COA566B2104 [24]	To evaluate the relative bioavailability of artemether–lumefantrine given as dispersible tablet for oral suspension, commercial tablet crushed for oral suspension and intact commercial tablet to healthy volunteers *N* = 48	Single dose of 4 dispersible tablets (20 mg artemether/ 120 mg lumefantrine) at once (intact or crushed) or one tablet of 80 mg artemether/ 480 mg lumefantrine	Artemether/DHA/lumefantrine: C_max_, T_max_, and AUC Rich PK sampling	Model development/update and validation (dispersible tablets) Model validation (crushed tablets)^a^
CCOA566B2303 [13]	To confirm the efficacy of the artemether–lumefantrine dispersible tablet in infants and children with a BW of ≥ 5 kg and < 35 kg suffering from *P. falciparum* malaria *N* = 889	≥ 5 kg – < 15 kg: 1 dispersible tablet ≥ 15 kg – < 25 kg: 2 dispersible tablets ≥ 25 kg – < 35 kg: 3 dispersible tablets One dispersible tablet contains 20 mg artemether/ 120 mg lumefantrine	Artemether/DHA: C_max_ on day 1 (1 h or 2 h) and T_max_ Lumefantrine: C_max_ on Day 3, T_max_ and C_168h_ Sparse PK Sampling: 1 and 2 h after 1st dose in patients before interim analysis; after interim analysis, 6 samples at different time points (6 h after dose 3, dose 5, dose 6, 24 h after dose 6 (day 3), day 7 and day 14)	Model development/update and validation
CCOA566B2306,EudraCT 2011–005852-33 [15]	To investigate the efficacy of artemether–lumefantrine during and following treatment with a 3-day regimen of artemether–lumefantrine dispersible tablet in infants < 5 kg of BW with acute uncomplicated *P. falciparum* malaria *N* = 20	One dispersible tablet (20 mg artemether/ 120 mg lumefantrine) was given twice daily for 3 consecutive days	Artemether/DHA: C_max_ on day 1 (1 h or 2 h) and T_max_ Lumefantrine: C_max_ on Day 3, T_max_ and C_168h_ Sparse PK sampling: 1 and 2 h after dose 1, 6 h after dose 5, 6 and 24 h after dose and any time on Day 7	Model validation
CALINA, NCT04300309, CCOA566B2307 [9]	To assess the key PK parameter of artemether in infants and neonates < 5 kg BW dosed with the new formulation of artemether–lumefantrine dispersible tablet (Cohort 1, infants; Cohort 2, neonates) *N* = 28	Two dispersible tablets (2.5 mg artemether/ 30 mg lumefantrine, i.e. 5 mg:60 mg) were given twice daily for 3 consecutive days	Artemether/DHA: C_max_ on day 1 (1 h or 2 h) and T_max_ Lumefantrine: C_max_ on Day 3, T_max_ and C_168h_ Samples 1, 2, 62, 66 and 168 h or 1, 2, 68, 84 and 168 h after dose 1	Model validation

^a^The PK of the crushed tablet arm of the study CCOA566B2104, was used as an additional validation as the dispersible tablet and crushed tablet had similar PK profiles

For all studies, all patients or legal guardians gave informed consent, and all studies followed the Declaration of Helsinki principles

### Model validation for artemether and lumefantrine PK

For comparison of PBPK model-predicted and observed artemether and lumefantrine C_max_ and lumefantrine C_168h_, populations were matched to those in the clinical trials for number of patients, age, weight, gender, and height, when available. These were important demographic variables needed to validate the PK prediction in malaria paediatric patients, as the age-related growth chart applied in the model from the ‘default’ Simcyp adults (Sim-Healthy Volunteers population) and paediatric patients (Sim-Paediatric population) was based on the North European Caucasian (NEC) population.

The default Sim-Healthy Volunteers virtual population was used for model validation of adult PK predictions. The default Sim-Paediatric population was used for model validation of the paediatric artemether and lumefantrine PK predictions.

In the Sim-Paediatric population, for CYP3A4-mediated metabolism of artemether and lumefantrine, the hepatic CYP3A4 ontogenies from Salem et al. [36] (Simcyp ontogeny ‘profile 1’) and Upreti and Wahlstrom [37] (Simcyp ontogeny ‘profile 2’) were used and evaluated to predict the PK of artemether and lumefantrine in infants and neonates from the CALINA study. The major difference between their proposed CYP3A4 ontogenies is in how they describe the CYP3A4 enzyme’s development from birth to adulthood. Salem et al. [36] suggest a more gradual increase in CYP3A4 expression starting low at birth and steadily increasing over time. Upreti and Wahlstrom [37] propose a sigmoidal growth pattern (CYP3A4 activity remains low in early life, then CYP3A4 matures quickly after infancy); see Additional File 2.

Based on the better ability of the CYP3A4 ontogeny ‘profile 2’ (Upreti and Wahlstrom, [37]) to predict the artemether and lumefantrine PK in the CALINA trial, this ontogeny was applied to predict neonate PK. The better predictive performance of the Upreti and Wahlstrom CYP3A4 ontogeny in pediatric patients is consistent with other reports [38–40].

The Simcyp default intestinal CYP3A4 [41] and hepatic CYP2B6 (Simcyp default ontogeny for CYP2B6 based on [42–44]) ontogenies were used.

Simulations of PK predictions in adults and paediatrics were conducted using custom trial design input (rather than age range and female ratio input only) in Simcyp according to the exact demographic data including individual age associated with BW and height from the trials summarized in Table 3.

### Model application

The artemether C_max_ after the first dose and lumefantrine C_max_ after the 6th dose and the concentration after 168 h (C_168h_) were predicted using the validated PBPK models of artemether and lumefantrine in neonates aged 1–28 days as well as for the three age subgroups of 1–7, 8–14, and 15–28 days. The simulations were conducted with *n* = 20 (5 patients × 4 trials), *n* = 100 (5 patients × 20 trials) and *n* = 1000 (100 patients × 10 trials). For validation and prediction of the population variability with the older neonates (aged 21–26 days) in the CALINA study, the selection of sample sizes of n = 20, *n* = 100, and *n* = 1000 in simulations was based on the available observed data between *n* = 5 to *n* = 22 from the CALINA study; *n* = 100, typically used with the Simcyp platform as a default population size, and *n* = 1000, representing a large pool of individuals in a virtual population. The approach was to capture the observed variability in infants and older neonates, as well as to assess the variability in a virtual neonate population.

Of note, the Simcyp paediatric PBPK model simulator can assess the impact of changing physiology over time by re-defining the patients over the simulation study period [45]. For lumefantrine, plasma concentrations at the end of 7 days (C_168h_) were investigated, which differed from artemether (*i.e.* C_max_ is assessed on Day 1), and the simulations were run using the re-defining patients over-time feature of Simcyp to predict C_max_ (after the 6th dose) and C_168h_. Accounting for time-based changes in physiology is particularly important in neonates who are rapidly growing and maturing in a short time frame. The sampling frequency of lumefantrine per age (days) was hourly for 0–3.5 days, 6-hourly for 3.5 to 6.5 days, 12-hourly for 6.5–14.2 days and daily for 14.2–30.3 days.

### Assumptions and uncertainties

Artemether is absorbed and cleared quickly with a T_max_ of 1.5–2 h [32]. In contrast, lumefantrine is absorbed and cleared more slowly (T_max_ of 6–8 h post-dose [32, 46]). Clinical studies [18, 47] reported that the PK of artemether and lumefantrine in adult healthy volunteers and malaria patients showed large interpatient variability. In the population PK (PopPK) analysis of artemether and lumefantrine [18] in adults with acute uncomplicated falciparum malaria, there was considerable interpatient variability in the absorption rate constant (ka) and bioavailability (F) of artemether and to a lesser degree for lumefantrine.

The incorporation of the variability in the PBPK model in this modelling and simulation study assumed the following: (1) source of variability resulting from the absorption process only; (2) same variability between adults and paediatrics for artemether; (3) in the absence of data indicating physiological changes in malaria patients, other default Simcyp CV% values (*i.e.,* 30%) remained unchanged. Additionally, the current models assume that the metabolism of artemether and lumefantrine by CYP3A were from CYP3A4 only, as the following parameter sensitivity analysis showed that the CYP3A5 and CYP3A7 do not appear to significantly impact the PK predictions in neonates and young infants.

### Parameter sensitivity analysis

Parameter sensitivity analyses (PSAs) were conducted by performing a series of simulations using the trial design in neonates from CALINA. The potential impact of neonate developmental changes on absorption and clearance, as well as the potential for CYP3A7 (mainly expressed in the foetal liver) involvement on predicted concentrations, was evaluated. A twofold range of lower and higher values than the current input values was used, based on all predictions being within twofold (0.5- to 2-fold) of the observed data across all age groups for the current input values.

Fraction absorbed (f_a_) and clearances of artemether and lumefantrine were evaluated by PSA to understand the sensitivity of these parameters on drug exposure. It is known that, particularly for lumefantrine, absorption is affected by food intake in neonates and infants, and bioavailability can influence clearance and in turn affect the predicted PK. The fraction absorbed (f_a_) for artemether in the current model was 1 (for neonates). The PSA was performed by varying artemether f_a_ values from 0.5 to 1 (twofold lower than the current setting of 1). Similarly, the PSA was also conducted by varying lumefantrine f_a_ values from 0.05 to 0.2 (twofold lower and higher than the current setting of 0.1). Additionally, the impact of clearance on the PK prediction was also conducted by PSA. For artemether, the range of intrinsic clearance was investigated using intrinsic CL_CYP3A4_/CL_CYP2B6_ from 1.135/6.10 µL/min/pmol CYP to 4.54/24.4 µL/min/pmol CYP. For lumefantrine, CL_CYP3A4_/CL_additional HLM clearance_ from 0.157/19.04 to 0.628/76.16 (µL/min/pmol CYP)/(µL/min/mg human liver microsome (HLM) protein) was used. These values were twofold lower and higher than the current PBPK model input values. Furthermore, to address the clinical impact of lumefantrine C_168h_ with respect to safety and efficacy due to combined effects of higher f_a_ and lower CL and lower f_a_ and higher CL, the simulations were conducted in neonates (n = 1000) aged from 1–28 days: (1) f_a_ = 0.05 and CL = twofold CL; (2) f_a_ = 0.2 and CL = 0.5-fold CL.

The potential impact of CYP3A7 on the overall metabolism of artemether and lumefantrine in infants and neonates was assessed using PSA. In the current models, the clearance of artemether and lumefantrine was assumed to be metabolized primarily by CYP3A4. The contribution of CYP3A7, which is mainly expressed in foetal liver [48] and declines in abundance after birth, was not considered in the PBPK model. It has been shown that CYP3A7 has a significantly lower metabolic capability compared to CYP3A4 based on the evaluation of the metabolism of 10 drug substrates in vitro [48]. By using a similar approach as reported by Zhou et al. [23], the PSA was conducted in older neonates (21–26 days, *n* = 5 as reported from CALINA) as well as in newborns aged 1–7 days, with 10 patients/10 trials, *n* = 100. The PSA was carried out by changing a value for CL_CYP3A7_ in the model, which ranged from 10 to 100% of CYP3A4 activity (*i.e.* range of 0.227 to 2.27 µL/min/pmol CYP3A7 and from 0.0314 to 0.314 µL/min/pmol CYP3A7 for artemether and lumefantrine, respectively).

Several publications [49, 50] have indicated that after birth, the CYP3A5 level showed no apparent increase as a function of age, only a very high degree of inter-individual variability. Therefore, a flat ontogeny of CYP3A5 is built into the Simcyp paediatric population, and the impact of any potential CYP3A5 clearance contributions was not evaluated by PSA.

## Results

### Model validation

The PBPK model validation in adult and paediatric patients for artemether C_max_, lumefantrine C_max_ and lumefantrine C_168h_ is summarized in Tables 4, 5, and 6, respectively. Overall, the predicted geometric mean values of the PK parameters and 90% confidence intervals (CI) were well predicted as they were all within twofold of the actual observed values in adults, as a defined criterion for successful PBPK model simulations for adults [20, 21] and for paediatric patients [22, 23].

**Table 4:** Model validation for artemether (Day 1) PK parameter prediction

Population	Trial	Dose, patient number (age range)	Geometric mean (90% CI)				Ratio (pred/obs)
			Observed		Predicted^a^		
			C_max_ ng/mL	AUC_0-6 h_ h*ng/mL	C_max_ ng/mL	AUC_0-6 h_ h*ng/mL	
Adults	B2104	80 mg single dose (SD), 4 × 20 mg dispersible tablets (DT) with food, *n* = 48 (22–50 years)	50.9 (44.7, 57.9)	168 (149, 188)	59.4 (50.0, 70.5)	270 (232, 315)	C_max_: 1.17 AUC: 1.61
Adults	B2104	80 mg SD 4 × 20 mg crushed tablets (CT) with food, *n* = 48 (22–50 years)	43.6 (39.2, 48.5)	156 (141, 173)	59.4 (50.0, 70.5)	270 (232, 315)	C_max_: 1.36 AUC: 1.73
Children BW > 5- ≤ 15 kg	B2303	20 mg b.i.d., 1 × 20 mg DT, *n* = 52 (0.3–5.68 years)	101 (73.4, 140)	---^b^	83.2 (69.6, 99.5)	---	0.82
> 15- ≤ 25 kg		40 mg b.i.d., 2 × 20 mg DT, *n* = 30 (3.78–10.7 years)	105 (77.3, 143)	---	105 (84.2, 131)	---	1.00
> 25- ≤ 35 kg		60 mg b.i.d., 3 × 20 mg DT, *n* = 9 (7.3–12.7 years)	112 (67.7, 184)	---	104 (79.6, 135)	---	0.93
Infants < 5 kg BW	B2306	20 mg b.i.d., 1 × 20 mg DT, *n* = 18 (37–215 days)	313 (154, 634)	---	250 (189, 331)	---	0.80
Infants < 5 kg BW	CALINA	5 mg b.i.d., 2 × 2.5 mg DT, *n* = 20 (53–157 days)	68 (45, 103)	---	63.5 (49.2, 81.9)	---	0.93
Neonates < 5 kg BW	CALINA	5 mg b.i.d., 2 × 2.5 mg DT, *n* = 5 (21–26 days)	62.2 (33.6, 115)	---	93.2 (62.4, 139)	---	1.50

*BW* body weight, *CI* confidence interval, *CT* crushed tablets, *DT* dispersible tablet, *obs* observed, *pred* predicted, *SD* single dose

^a^All simulations were conducted according to the number of patients, gender, BW, and height (if available) in the individual BW and age range from trials

^b^–-, not available

**Table 5:** Model validation for lumefantrine (Day 3) PK parameter prediction

Population	Trial	Dose, subject number (age range)	Geometric mean (90% CI)				Ratio (pred/obs)
			Observed		Predicted^a,b^		
			C_max_ (D3) µg/mL	AUC_inf_ h*µg/mL	C_max_ (D3) µg/mL	AUC_inf_ h*µg/mL	
Adults	B2104	480 mg single dose (SD), 4 × 120 mg dispersible tablets (DT) with food, *n* = 48 (22–50 years)	9.52 (8.87, 10.2) (Day1)	261 (238, 287)	10.6 (8.98, 12.5) (Day 1)	288 (257, 323)	Cmax: 1.11 AUC: 1.10
Adults	B2104	480 mg SD 4 × 120 mg crushed tablets (CT) with food, *n* = 48 (22–50 years)	10.5 (9.82, 11.2)	295 (271, 321)	10.6 (8.98, 12.5) (Day 1)	288 (257, 323)	Cmax: 1.01 AUC: 0.98
Children BW > 5- ≤ 15 kg	B2303	120 mg bid, 1 × 120 mg DT, *n* = 52 (0.3–5.68 years)	3.85 (3.36, 4.42)	---^c^	2.39 (2.20, 2.60)	---	0.62
> 15- ≤ 25 kg		240 mg bid, 2 × 120 mg DT, *n* = 30 (3.78–10.7 years)	6.59 (5.60, 7.75)	---	6.48 (5.75, 7.31)	---	0.98
> 25- ≤ 35 kg		360 mg bid, 3 × 120 mg DT, *n* = 9 (7.3–12.7 years)	8.34 (5.64, 12.3)	---	8.01 (6.19, 10.4)	---	0.96
Infants < 5 kg BW	B2306	120 mg bid, 1 × 120 mg DT, *n* = 19 (37–215 days)	5.17 (3.73, 7.15)	---	5.97 (4.78, 7.44)	---	1.15
Infants < 5 kg BW	CALINA	60 mg bid, 2 × 30 mg DT, *n* = 22 (53–157 days)	3.18 (2.53, 4.00)	---	2.90 (2.42, 3.47)	---	0.91
Neonates < 5 kg BW	CALINA	30 mg bid, 2 × 30 mg DT, *n* = 5 (21–26 days)	4.65 (3.31, 6.53)	---	4.60 (3.21, 6.59)	---	0.99

*BW* body weight, *CI* confidence interval, *CT* crushed tablets, *DT* dispersible tablet, *obs* observed, *pred* predicted, *SD* single dose

^a^All simulations were conducted according to the number of subjects, gender, BW, and height (if available) in the individual BW and age range from trials

^b^f_a_ was entered at 0.1 for 60 and 120 mg, 2 × f_a_ = 0.2 for dose regimen of 240 mg and 360 mg and f_a_ was 1 for 480 mg SD in adults

^c^–-, not available

**Table 6:** Model validation for lumefantrine concentration at 168 h (C_168h_) prediction

Population	Trial	Dose, patient number (age range)	Geometric mean (90% CI)		Ratio (pred/obs)
			Observed ^a^	Predicted ^b,c^	
			C_168h_ ng/mL	C_168h_ ng/mL	
Children BW > 5- ≤ 15 kg	B2303	120 mg bid, 1 × 120 mg DT, *n* = 27 (0.3–5 years)	212 (156, 287)	344 (290, 408)	1.62
> 15- ≤ 25 kg		240 mg bid, 2 × 120 mg DT, *n* = 4 (3–5 years)	388 (170, 887)	565 (376, 850)	1.46
> 25- ≤ 35 kg		360 mg bid, 3 × 120 mg DT	---^d^	---	---
Infants < 5 kg BW	B2306	120 mg bid, 1 × 120 mg DT, *n* = 16 (37–215 days)	666 (500, 888)	876 (678, 1133)	1.32
Infants < 5 kg BW	CALINA	60 mg bid, 2 × 30 mg DT, *n* = 22 (53–157 days)	353 (250, 498)	471 (382, 582)	1.33
Neonates < 5 kg BW	CALINA	60 mg bid, 2 × 30 mg DT, *n* = 5 (21–26 days)	615 (403, 937)	803 (527, 1223)	1.31

*BW* body weight, *CI* confidence interval, *DT* dispersible tablet, *obs* observed, *pred* predicted

^a^Formulation was dispersible tablet

^b^All simulations were conducted according to the number of patients, gender, BW, and height (if available) in the individual BW and age range from trials

^c^f_a_ was entered at 0.1 for 60 and 120 mg, 2 × f_a_ = 0.2 for dose regimen of 240 mg

^d^–-, not available

### Paediatric artemether and lumefantrine PBPK model validation: a comparison of simulated versus observed artemether–lumefantrine PK in paediatric patients

In the present PBPK model validation, models with two different ontogenies of hepatic CYP3A4 were evaluated: (1) hepatic CYP3A4 ontogeny reported by Upreti and Wahlstrom [37], and (2) according to the ontogeny published by Salem et al. [36]. The simulations of the CALINA study showed an overprediction of C_max_ in infants and older neonates when the Salem hepatic CYP3A4 ontogeny (‘profile 1’) was used. The model using Upreti hepatic CYP3A4 ontogeny (‘profile 2’) performed better in predicting the observed artemether and lumefantrine concentrations in paediatric patients (Additional File 3).

Furthermore, a series of simulations for lumefantrine C_max_ and C_168h_ to compare with values from CALINA were performed using no-, slow-, medium- and fast-ontogenies for the remaining non-CYP3A4 metabolic clearance assigned as an additional HLM metabolic clearance of lumefantrine. The prediction results indicated that having no ontogeny (*i.e.* at 100% of adult levels) in the current model resulted in the best prediction relative to the observed clinical data. Hence, this was selected for model application.

In the CALINA study, a dose of 5 mg artemether and 60 mg lumefantrine was administered to infants (< 5 kg BW aged 53–157 days) and neonates (< 5 kg BW aged 21–26 days). The model-predicted geometric mean values and 90% confidence intervals (90% CI) versus the observed values for artemether C_max_ on Day 1, lumefantrine C_max_ on Day 3, and C_168h_ are shown in Additional File 4. The model predicted the geometric mean artemether C_max_, lumefantrine C_max_ and C_168h_ in infants and older neonates within ≤ 1.5-fold of the observed values.

Additionally, the model was validated by predicting plasma concentrations of artemether and lumefantrine and comparing them with historical values for CCOA566B2303 [13, 14] and CCOA566B2306 (EudraCT 2011-005852-33). In CCOA566B2303, a fixed dose of artemether/lumefantrine 20/120 mg, artemether/lumefantrine 40/240 mg, and artemether/lumefantrine 60/360 mg was administered to children in three BW bands of > 5 to  ≤ 15 kg, > 15 to  ≤ 25 kg and > 25 to  ≤ 35 kg, respectively. Because lumefantrine showed a positive food effect, the current lumefantrine model assumed f_a_ of 0.1 (milk intake) and 0.2 (light meal) for children with BW of > 5 to  ≤ 15 kg and > 15 to  ≤ 35 kg, respectively. For children aged 0.3–5 years and BW > 5 to  ≤ 15 kg, the food intake may not have been limited to milk, which is hypothesized to have been a potential cause of the under-predicted absorption in this group, shown in Additional File 5 for the prediction of lumefantrine C_max_ in trial CCOA566B2303, with a prediction/observed ratio of 0.62. However, the prediction error was still within twofold of observed values. In study CCOA566B2306, a fixed dose of artemether/lumefantrine 20/120 mg was administered to infants with BW of < 5 kg. The simulated and observed artemether and lumefantrine concentrations were also compared. The model-predicted geometric mean artemether C_max_, lumefantrine C_max_ and C_168h_ in infants are within twofold of the observed values.

### Model application using a virtual population

**Fig. 2:**
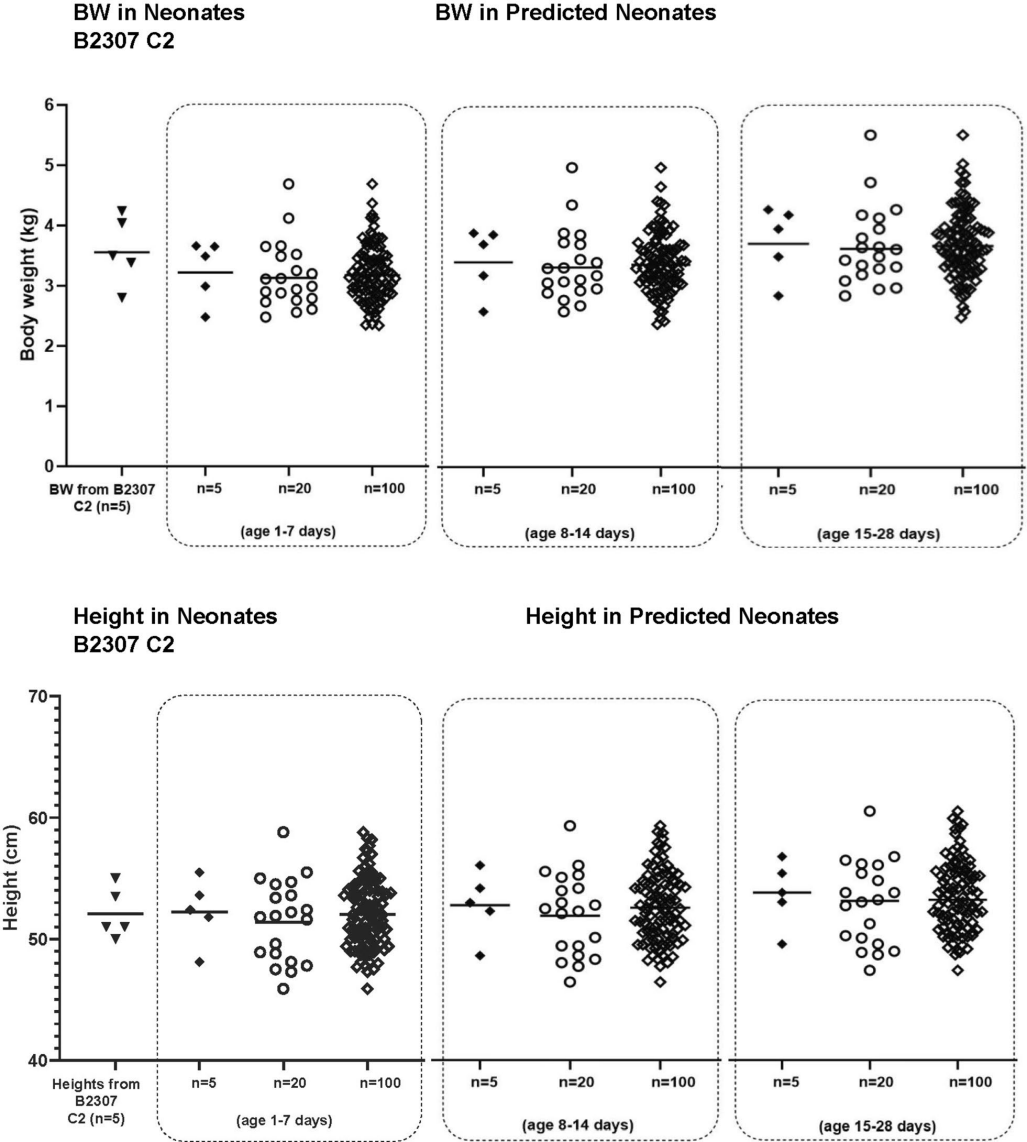
Comparison of body weights and heights of predicted neonates and neonates from CALINA. Body weights and heights of neonates 15–28 days, 8–14 days and 1–7 days of age, using Simcyp default paediatric population to neonates from CALINA. B2307: CALINA study, C2: Cohort 2

The artemether and lumefantrine models were applied to predict artemether C_max_ values after the 1st dose of 5 mg artemether, and lumefantrine C_max_ and C_168h_ values after the 6th dose of 60 mg lumefantrine (b.i.d.) in neonates 1–28 days of age in order to assess the potential variability that might be observed clinically. The age group of 1–28 days was split into three subgroups of 1–7, 8–14 and 15–28 days. The Simcyp default virtual paediatric population was used. The comparison of BW and height from simulation output files for each of the three subgroups to the observed data in older neonates from CALINA is shown in Fig. 2. These patient characteristics from the CALINA study were well captured by the Simcyp default virtual paediatric population.

Using the virtual populations of n = 20, 100, or 1000, the predicted median with 95th and 5th percentiles of between-patient variability were compared to the observed clinical data in infants and older neonates (e.g. 21–26 days) to assess if the PBPK models capture the clinically observed variability with the predicted individual values.

PBPK model predictions were conducted using the Simcyp paediatric population with default demographics for ages 1–7 days, 8–14 days, or 15–28 days and female ratio = 0.5, for *n* = 20 (5 patients × 4 trials), *n* = 100 (5 patients × 20 trials) and *n* = 1000 (100 patients × 10 trials).

### *Artemether C*_*max*_* predictions in neonates* < *5 kg BW using a virtual population*

**Fig. 3:**
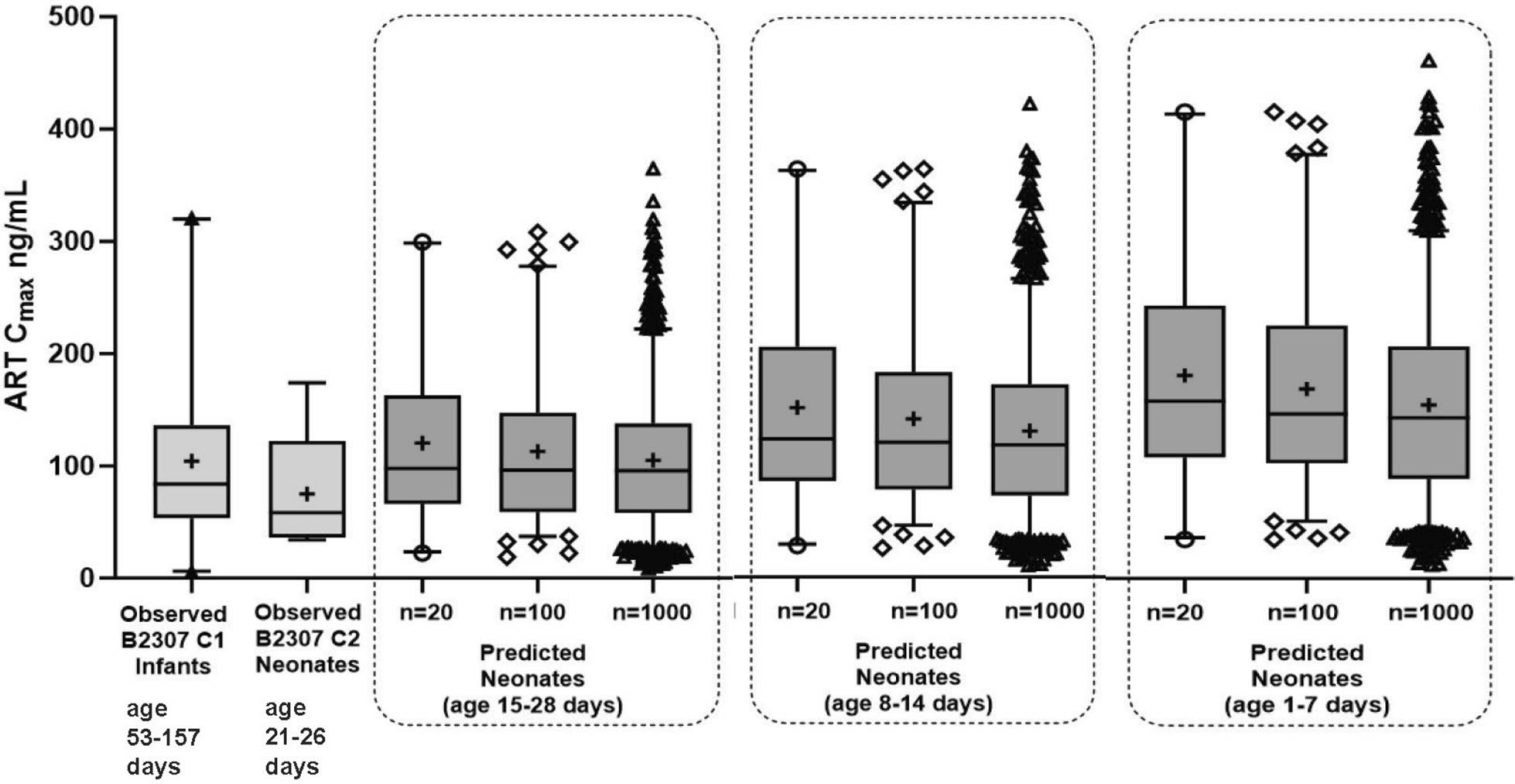
Variability of predicted and observed artemether C_max_ post first dose. Legend: variability of predicted artemether C_max_ post first dose in neonates age 15–28 days, 8–14 days and 1–7 days compared to the variability of the observed artemether C_max_ in infants and neonates in the CALINA study. B2307: CALINA study, C1: Cohort 1, C2: Cohort 2

The predicted individual patient numbers of *n* = 20, 100, or 1000 for artemether C_max_ are summarized in Additional File 5. The predicted variability in neonates is comparable or higher than the clinically observed data (Fig. 3). The highest predicted artemether C_max_ values are within the range of safe and efficacious concentrations observed in clinical studies in adult and paediatric patient populations. As shown in Additional file 6, predicted exposures are also comparable to those observed in paediatric patients of >5 kg body weight from Study COA566B2303 [13] (as were those of lumefantrine C_max_).

### *Lumefantrine C*_*168h*_* predictions in neonates* < *5 kg BW using a virtual population*

**Fig. 4:**
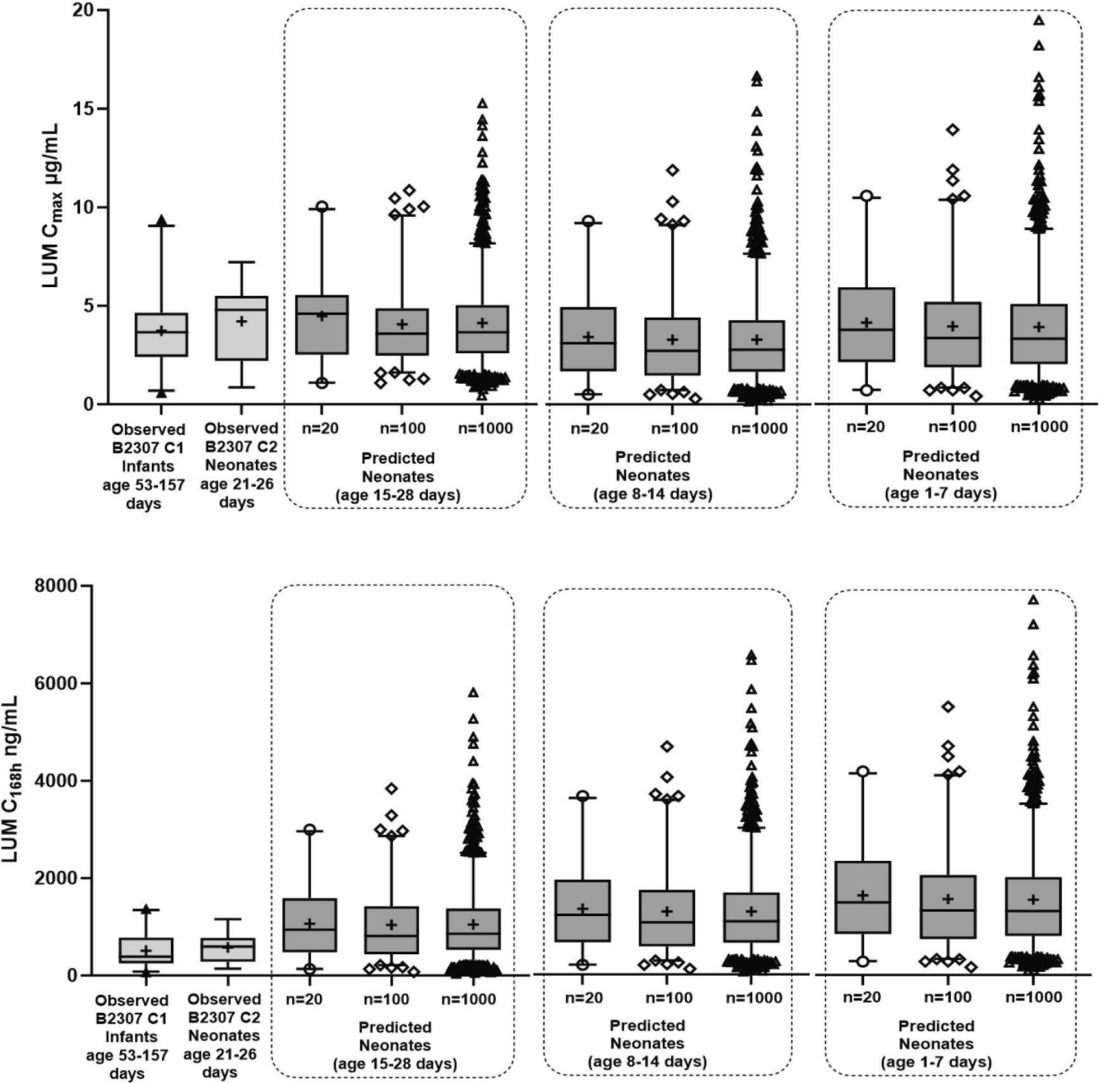
Variability of the predicted and observed lumefantrine C_max_ post 6th dose and C_168h_. Legend: variability of the predicted lumefantrine C_max_ post 6th dose and C_168h_ in neonates of age 15–28 days, 8–14 days and 1–7 days compared to the observed variability of lumefantrine C_max_ and C_168h_ in infants and neonates

The predicted lumefantrine C_168h_ in neonates for *n* = 20, 100, or 1000 patients is summarized in Additional File 7. The predicted variability in neonates was comparable to or higher than observed data (Fig. 4). The predicted lumefantrine C_168h_ 5th percentiles were above the efficacy marker for exposure, *i.e*. 200 ng/mL, which is within the range of safe and efficacious concentrations observed in clinical studies.

### Parameter sensitivity analyses

Simulations were conducted using the exact number of patients and patient characteristics, *i.e.* age, gender, BW, and heights in neonates from the CALINA study.

### Impact of absorption and clearance on the prediction of neonate artemether and lumefantrine pharmacokinetics

As shown in Additional File 8, varying artemether f_a_ from 1 (the current model) to 0.5 (twofold lower) would result in a decrease in predicted geometric mean of artemether C_max_ from 93.2 ng/mL to 58.1 ng/mL (closer to the observed value of 62.2 ng/mL), respectively. PSA was also performed on artemether clearance (Additional File 8, lower graph) to determine if the prediction of artemether C_max_ was similarly sensitive to changes in the artemether f_a_ as to changes in artemether clearance. Increases of artemether clearance to ~ twofold of the final PBPK model input resulted in a C_max_ value of 59.3 ng/mL (closer to the observed value of 62.2 ng/mL). These PSA results suggested similar sensitivities in f_a_ and clearance to impact artemether C_max_, *i.e.* similar changes in absorption or clearance parameters of artemether would result in a similar impact on the predicted maximal plasma concentrations. The over-predicted geometric mean artemether C_max_ of 93.2 ng/mL versus the observed value of 62.2 ng/mL (of the 5 neonates in the study) may be due to the small sample size and potential variability in artemether absorption and/or clearance.

For lumefantrine C_168h_, whereas a twofold change in f_a_ above and below the input value of 0.1 appears to be linear (Additional File 9, top graph), increasing clearance twofold results in an over-proportional decrease in C_168h_ (Additional File 9, lower graph). This PSA suggests that a small increase in clearance in the model may improve the prediction of the C_168h_ value (predicted lumefantrine C_168h_ 803 ng/mL versus observed 615 ng/mL). However, lumefantrine C_max_ at Day 3 was predicted well with current model inputs for f_a_ and clearance.

### Impact of CYP3A7 contribution on the overall metabolism of artemether and lumefantrine

A sensitivity analysis was conducted in neonates (21–26 days from CALINA) and newborns (1–7 days) using CL_CYP3A7_ ranging from 0.227 to 2.27 µL/min/pmol CYP and from 0.0314 to 0.314 µL/min/pmol CYP (10% to 100% of CL_CYP3A4_) for artemether and lumefantrine, respectively. As shown in Additional File 10, across the range of values investigated versus relative predicted, changes in plasma artemether or lumefantrine concentrations in older neonates were minimal, suggesting that CYP3A7 plays a minimal role in artemether or lumefantrine metabolism/elimination in older neonates in the age range evaluated (21–26 days). As shown in Additional File 11, across the range of values investigated versus relative predicted, change in plasma artemether or lumefantrine concentrations in neonates (1–7 days) would result in a decrease of predicted geometric mean of artemether C_max_ and lumefantrine C_168h_ by ~ 17% and 14%, respectively, when 50% of contribution of CYP3A7 to the overall metabolism of CYP3A-mediated pathways was assumed. In a worst-case scenario, when 100% of CYP3A7 contributed to the CYP3A-mediated pathways, the predicted artemether and lumefantrine concentrations would decrease less than 50% as compared with the predicted values in the current model.

## Discussion

The CALINA study is one of the first to evaluate antimalarial treatment in patients of < 5 kg BW with uncomplicated *P. falciparum* neonatal malaria, a little-studied patient population. It assessed an optimized dispersible tablet formulation of artemether–lumefantrine to provide a dose of 5 mg artemether and 60 mg lumefantrine, developed for use in infants < 5 kg BW. Pharmacokinetic evaluations in the CALINA study demonstrated that artemether and lumefantrine exposures in infants < 5 kg BW treated with this initial optimized formulation at a dose of artemether/lumefantrine 5/60 mg were within the safe and efficacious range observed following treatment with the currently available dispersible tablet (20/120 mg) in older paediatric patients. Due to the low recruitment of neonates (aged < 28 days), a validated PBPK model was further developed and refined, then used in infants (> 28 days and < 5 kg) and neonates (≤ 28 days and < 5 kg) to predict doses that could achieve the safe and efficacious exposures observed previously in paediatric patients of ≥ 5 kg BW. The key PK parameter that we focused on was artemether C_max_ (after the first dose). This parameter is associated with early parasite clearance and the potential to cause neurotoxicity at higher concentrations, the latter based on preclinical evidence with prolonged systemic exposures following intramuscular exposure in dogs [51]. Artemether C_max_ is also the primary endpoint of the CALINA study. The other main focus of the study was lumefantrine C_168h_, which is an accepted marker for 28-day cure rate [30, 52, 53]. Given the limitations in the number of PK blood samples in the CALINA study, with only a small number of sampling points, other parameters, for example lumefantrine AUC, could not be calculated. However, AUCs for both artemether and lumefantrine were verified using adult data from Study COA566B2104 [24].

The elimination of artemether and lumefantrine is estimated to be at least 50% mediated by CYP3A. Simulations of both artemether and lumefantrine models were evaluated using two different ontogenies of hepatic CYP3A4 [36, 37] in the paediatric population. The model using Upreti hepatic CYP3A ontogeny [37] appeared to perform better in predicting the observed values in infants and neonates.

Model-predicted exposures of artemether and lumefantrine in neonates (1–28 days) were within the range of safe and efficacious exposures observed in paediatric patients of ≥ 5 kg and within twofold of observed exposures (≥ 5 kg BW). Overall, the comparison of observed and predicted concentrations with variability was within the prediction error of < twofold. The validated model was used to predict plasma C_max_ values for artemether and lumefantrine and lumefantrine C_168h_ in virtual neonate patients with high confidence. Model-predicted variability in infants and neonates aged 15–28 days, 8–14 days, and 1–7 days was comparable to or larger than the variability of the observed artemether and lumefantrine concentrations in infants and the older neonates in CALINA, giving us additional confidence in the simulations. Although the CALINA study included a relatively small number of patients, potentially limiting assessment of safety, model predictions of artemether C_max_ and lumefantrine C_max_ values using 100 and 1000 virtual patients provide reassurance about use of the 5 mg: 60 mg artemether–lumefantrine b.i.d. dosing regimen for 3 days in infants and neonates with uncomplicated falciparum malaria, as the predicted exposures are comparable to those observed in paediatric patients of > 5 kg body weight from study COA566B2303 [13].

Potential limitations of the study include not taking into account the induction potential of artemether on CYP2B6 in the model, and hence auto-induction, as previously reported [54, 55]. Nevertheless, the impact of auto-induction on the predicted artemether C_max_ after the first dose is expected to be minimal because the process of CYP enzyme synthesis takes time to manifest significant changes in the metabolism and hence exposure of artemether and requires multiple doses.

Also, the contributions of CYP3A5 and CYP3A7 were not considered in the model development. CYP3A7 is mainly expressed in foetal liver [48]; therefore, sensitivity analyses conducted in neonates (21–26 days from CALINA) and newborns (1–7 days) were performed and showed the contribution of CYP3A7 to the overall clearance of artemether and lumefantrine may not have a substantial impact for neonates. CYP3A5 is polymorphically expressed between and within ethnic groups and is more frequently expressed in the livers of Africans than in those of Caucasians [56]. The clinical trial participants used for the model validation were of African (CCOA566B2303, CCOA566B2306, CALINA) and Caucasian (CCOA566B2104) origin. The models predicted the observed plasma concentrations in both ethnic groups across all age groups within twofold of the observed data; these results were in line with publications [57]. The polymorphic distribution of the wildtype CYP3A5*1 allele indicates that metabolically active CYP3A5 is expressed in an estimated 30% of Caucasians and more than 50% of Africans [56]. CYP3A5*3 is the most frequently recognized nonfunctional allele and one of the most frequent polymorphisms [57]. In Caucasians, the frequency of CYP3A5*3 has been shown to be ≥ 90%, whereas the occurrence among black Africans ranges from 11 to 78% [58]. A recent review article [60] suggests a lack of substantial influence of polymorphisms in CYP enzymes, such as CYP3A4 and CYP3A5. Furthermore, a population PK study carried out in Tanzania [60] indicated that CYP3A5*3 did not affect lumefantrine plasma concentrations significantly as compared to the wildtype (CYP3A5*1). Similar findings were observed for artemether, whereby patients with CYP3A5*3 did not have different plasma concentration values compared to the wildtype [59]. Despite the potential inter-ethnic variability in protein expression of CYP3A5 alleles, the PBPK model can reasonably describe artemether and lumefantrine exposure.

## Conclusions

This is one of the first times that PBPK modelling has been used to inform the dosing recommendation in neonates. Exposures predicted with the PBPK modelling supplemented the observed data from CALINA to support the recommendation of use of artemether/lumefantrine 5/60 mg b.i.d. for 3 days for neonates and young infants with uncomplicated malaria. Based on the success of the PBPK models for artemether and lumefantrine in predicting drug concentrations in adults and children, including neonates, modelling and simulation were used with confidence to supplement the limited available data for neonates < 5 kg BW obtained from the CALINA clinical study for this difficult-toto-recruit patient population. This study has demonstrated that PBPK modelling is able to enhance confidence in the overall conclusion.

## Supplementary Information

Additional file 1. PBPK model for dihydroartemisinin

Additional file 2. CYP3A4 ontogenies proposed by Salem and Upreti

Additional file 3. Impact of CYP3A4 ontogeny profiles on the simulated PK parameters for artemether and lumefantrine in infants and neonates (<5 kg body weight).

Additional file 4. Model predicted geometric mean values and 90% confidence intervals (90% CI) versus the observed values for artemether C_max_ on Day 1, lumefantrine C_max_ on Day 3, and lumefantrine C_168h_.

Additional file 5. Predicted artemether maximum plasma concentrations (Day 1) in neonates after a first dose of 5 mg artemether + 60 mg lumefantrine.

Additional file 6. Model-predicted artemether and lumefantrine C_max_ compared with observed data from the CALINA study and Study COA566B2303.

Additional file 7. Predicted lumefantrine C_168h_ in neonates for n=20, 100, or 1000 patients.

Additional file 8. Parameter sensitivity analyses for absorption and clearance input parameters on the predicted plasma concentrations for artemether.

Additional file 9. Parameter sensitivity analyses for absorption and clearance input parameters on the predicted plasma concentrations for lumefantrine.

Additional file 10. Parameter sensitivity analysis of CYP3A7 contributions to the overall metabolism on the predicted plasma concentrations in neonates: potential impact of CYP3A7 contributions on older neonates (21-26 days, n=5).

Additional file 11. Parameter sensitivity analysis of CYP3A7 contributions to the overall metabolism on the predicted plasma concentrations in neonates: potential impact of CYP3A7 contributions on newborns (1-7 days, n=100).

## Data Availability

The sponsor of this study is committed to sharing with qualified external researchers, access to patient-level data and supporting clinical documents from eligible studies. These requests are reviewed and approved by an independent review panel on the basis of scientific merit. All data provided are anonymized to respect the privacy of patients who have participated in the trial in line with applicable laws and regulations. This trial data availability is according to the criteria and process described on www.clinicalstudydatarequest.com.
