# Supplementary material for: Physiologically based pharmacokinetic modelling to predict artemether and lumefantrine exposure in neonates weighing less than 5 kg treated with artemether–lumefantrine to supplement the clinical data from the CALINA study
Source: Trop Med Health. 2025 Aug 25;53:116. doi: 10.1186/s41182-025-00790-w (PMC12376358; doi:10.1186/s41182-025-00790-w)
Supplement: Supplementary file 1 — Additional file 1. PBPK model for dihydroartemisinin [file 41182_2025_790_MOESM1_ESM.pdf]

Helen Gu et al. Physiologically-based pharmacokinetic modeling to predict artemether and lumefantrine exposure in neonates weighing less than 5 kg treated with artemether-lumefantrine to supplement the clinical data from the CALINA study

Additional file 1: PBPK model for dihydroartemisinin

The PBPK model of dihydroartemisinin (DHA) was developed, validated and applied to PK predictions in the pediatric population.

The DHA model was adapted from a Simcyp compound file (version 19) from the Simcyp global health repository and Abla et al (2023). The parameters of V_{ss} and CL/F were modified to 0.63 L/kg and 144 L/h, respectively, to fit the observed data in adults (B2104). The input parameters are described in the following table.

PBPK model input parameters for artemether metabolite dihydroartemisinin

Parameter (unit)	Value	Source
Physicochemical and plasma binding		
Molecular weight (g/mol)	284.35	
logP	2.3	
Compound type	Neutral	
B/P	1	
Fup	0.105	
Main binding protein	AGP	Assumption
Distribution (full PBPK)		
V_{ss} (L/kg)<br>Adults (pediatrics)	0.63	Full PBPK “method 2” predicted with Kp scalar 0.65.<br>Optimized to recover DHA in adults (B2104-DT)
Elimination		
CL_{po} (L/h)<br>Adults	144	CL/F =2.76 L/h/kg with mean BW of 52.1 kg in adults
CLr (L/h)	0	No and insignificant renal excretion found in ART, LUM or DHA<br>Coartem prescribing information (Novartis)

The DHA model was developed/updated based on the adult data CCOA566B2104 (dispersible tablet) and validated for PK predictions using clinical data from CCOA566B2104 (dispersible and crushed tablets), CCOA566B2303, CCOA566B2306 and CCOA566B2307. DHA is mainly metabolized by UGT1A9 and UGT2B7 (Ilett et al. 2002). However, the DHA model that was used did not include the contributions of these enzymes, as there may be some uncertainty in the ontogeny of UGT enzymes. Instead, the *in vivo* clearance in the adult model was based on the PK analysis after oral and intravenous administrations of artemether in adults (Silamut et al 2003), and was validated and applied for pediatric DHA C_{max} predictions

The simulations of ART (parent) and DHA (metabolite) was conducted simultaneously using the same custom trial design populations in Simcyp according to the exact demographic data from trials CCOA566B2104, CCOA566B2303, CCOA566B2306 and CCOA566B2307.

The summary of comparison of the predicted versus observed values is listed in the following table. The model validation results showed the prediction errors (predicted value/observed value) are within 2-fold (>0.5- <2).

Simulated and observed historical C_{max} on Day 1 for DHA across adult and paediatric populations

PK parameter	Age, year (BW, kg)	Trial (Dose, mg)	Observed	Predicted	Pred/Obs
C_{max}, ng/mL Geometric mean (90% CI)	Adults 22-50	B2104 dispersible tablet (80 mg)	52.7 (47.7, 58.2) (n=48)	51.4 (43.5, 60.8) (n=48)	0.98
		B2104 crushed tablet (80 mg)	46.4 (42.0, 51.2) (n=48)	51.4 (43.5, 60.8) (n=48)	1.11
	0 - 5 (≥5 - ≤15)	B2303 (20 mg)	31.7 (23.2, 43.5) (n=52)	48.8 (40.8, 58.4) (n=52)	1.54
	4 - 8 (≥15 - ≤25)	B2303 (40 mg)	45.0 (32.6, 62.2) (n=30)	75.5 (58.1, 98.2) (n=30)	1.68
	7 – 12 (≥25 - ≤35)	B2303 (60 mg)	57.5 (34.3, 96.4) (n=9)	73.5 (45.0, 120) (n=9)	1.28
	37 – 214 days (≥2.57 - ≤4.93)	B2306 (20 mg)	76.3 (49.7, 119)	80.1 (59.6, 108) (n=18)	1.05
	Infants (<5 kg)	B2307 (5 mg)	11.5 (7.58, 17.4) (n=20)	21.2 (15.9, 28.2) (n=20)	1.84
	Neonates (<5 kg)	B2307 (5 mg)	15.7 (8.53, 28.9) (n=5)	23.1 (11.5, 46.2) (n=5)	1.47

The DHA model was then applied to simulate DHA C_{max} values after the 1st dose (Day 1) of 5 mg artemether in neonates aged 1-28 days. The age group of 1-28 days was split into three subgroups of 1-7 days, 8-14 days and 15-28 days. The Simcyp default pediatric population was used. The predicted individual subjects of n=20, n=100, and n=1000 for DHA C_{max} is summarized in the following table.

Predicted DHA maximum plasma concentrations (Day 1) in neonates after first dose of 5 mg artemether and 60 mg

Age range (day)	BW (kg) Mean ± SD	Plasma concentration DHA C_{max}, ng/mL (1st dose on Day 1)		
		Mean ± SD	Median (range)	Geometric mean (90% CI)
1 - 28 (n=20)	3.51 ± 0.66	26.6 ± 19.6	18.9 (8.20, 72.2)	21.3 (16.5, 27.5)
1 - 28 (n=100)	3.53 ± 0.57	26.9 ± 19.0	21.6 (1.87, 93.7)	20.7 (18.2, 23.6)
1 - 28 (n=1000)	3.55 ± 0.55	24.2 ± 18.5	19.5 (0.86, 119)	17.7 (16.9, 18.5)
1 - 7 (n=20)	3.17 ± 0.55	20.5 ± 14.4	14.1 (7.94, 55.5)	16.8 (13.3, 21.4)
1 - 7 (n=100)	3.20 ± 0.45	21.0 ± 14.4	16.8 (1.76, 73.9)	16.6 (14.7, 18.7)
1 - 7 (n=1000)	3.25 ± 0.45	19.4 ± 14.7	15.7 (0.65, 105)	14.5 (113.9, 15.2)
8 -14 (n=20)	3.37 ± 0.59	24.3 ± 17.0	17.8 (9.40, 64.1)	20.4 (16.1, 25.8)
8 -14 (n=100)	3.41 ± 0.49	25.1 ± 16.9	20.1 (2.52, 87.1)	20.0 (17.8, 22.5)
8 - 14 (n=1000)	3.45 ± 0.49	22.9 ± 16.7	18.7 (0.83, 114)	17.4 (16.7, 18.2)
15 - 28 (n=20)	3.68 ± 0.65	27.9 ± 19.2	20.7 (10.1, 73.1)	23.1 (18.2, 29.2)
15 - 28 (n=100)	3.71 ± 0.55	28.3 ± 18.9	22.7 (2.94, 94.8)	22.6 (20.1, 25.4)
15 - 28 (n=1000)	3.76 ± 0.54	25.8 ± 18.4	20.8 (0.98, 120)	19.6 (18.8, 20.4)

The simulations were conducted in a population across 4 different age ranges with 5 subjects/4trials (n=20), 5 subjects/20 trials (n=100), 100 subjects/10 trials (n=1000). The female ratio was 0.5.

References

Abla N, Howgate E, Rowland-Yeo K, et al (2023) Development and application of a PBPK modeling strategy to support antimalarial drug development. CPT Pharmacometrics Syst Pharmacol;12:1335–46.

Ilett KF, Ethell BT, Maggs JL, et al (2002). Glucuronidation of dihydroartemisinin in vivo and by human liver microsomes and expressed UDP-glucuronosyltransferases. Drug Metab Dispos;30(9):1005-12.

Silamut K, Newton PN, Teja-Isavadharm P, et al (2003). Artemether bioavailability after oral or intramuscular administration in uncomplicated falciparum malaria. Antimicrob Agents Chemother;47(12):3795-8.
