# Supplementary material for: Physiologically based pharmacokinetic modelling to predict artemether and lumefantrine exposure in neonates weighing less than 5 kg treated with artemether–lumefantrine to supplement the clinical data from the CALINA study
Source: Trop Med Health. 2025 Aug 25;53:116. doi: 10.1186/s41182-025-00790-w (PMC12376358; doi:10.1186/s41182-025-00790-w)
Supplement: Supplementary file 2 — Additional file 2. CYP3A4 ontogenies proposed by Salem and Upreti [file 41182_2025_790_MOESM2_ESM.pdf]

Helen Gu et al. Physiologically-based pharmacokinetic modeling to predict artemether and lumefantrine exposure in neonates weighing less than 5 kg treated with artemether-lumefantrine to supplement the clinical data from the CALINA study

Additional File 2: CYP3A4 ontogenies proposed by Salem and Upreti

Hepatic CYP3A4 from 0-18 years

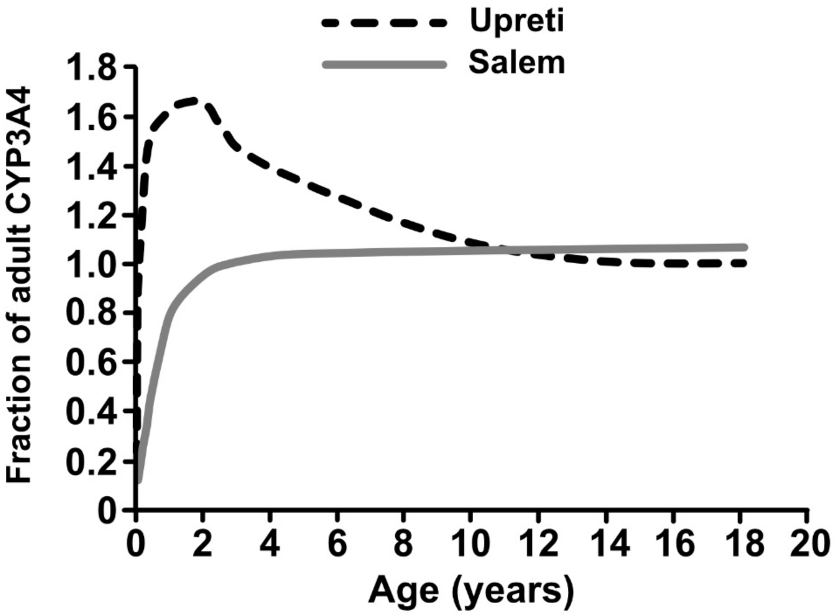

Hepatic CYP3A4 from 0-365 days

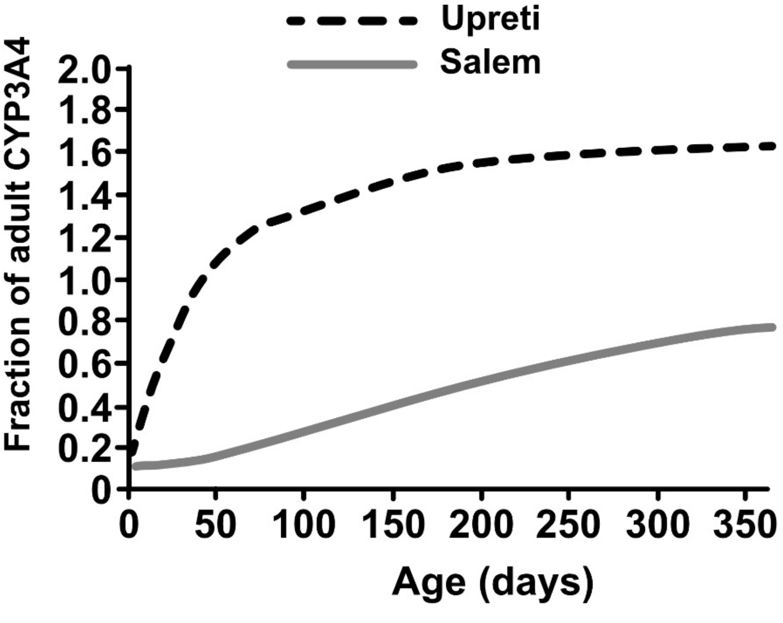

Salem ontogeny: Salem F, Johnson TN, Abduljalil K, et al. A Re-evaluation and Validation of Ontogeny Functions for Cytochrome P450 1A2 and 3A4 Based on In Vivo Data. Clin Pharmacokinet. 2014; 53(7):625-36.
Upreti ontogeny: Upreti VV, Wahlstrom JL. Meta-analysis of hepatic cytochrome P450 ontogeny to underwrite the prediction of pediatric pharmacokinetics using physiologically based pharmacokinetic modeling. J Clin Pharmacol. 2016; 56(3), 266-83.
