# Supplementary material for: Physiologically based pharmacokinetic modelling to predict artemether and lumefantrine exposure in neonates weighing less than 5 kg treated with artemether–lumefantrine to supplement the clinical data from the CALINA study
Source: Trop Med Health. 2025 Aug 25;53:116. doi: 10.1186/s41182-025-00790-w (PMC12376358; doi:10.1186/s41182-025-00790-w)
Supplement: Supplementary file 3 — Additional file 3. Impact of CYP3A4 ontogeny profiles on the simulated PK parameters for artemether and lumefantrine in infants and neonates (<5 kg body weight). [file 41182_2025_790_MOESM3_ESM.pdf]

Helen Gu et al. Physiologically-based pharmacokinetic modeling to predict artemether and lumefantrine exposure in neonates weighing less than 5 kg treated with artemether-lumefantrine to supplement the clinical data from the CALINA study

Additional file 3: Impact of CYP3A4 ontogeny profiles on the simulated PK parameters for artemether and lumefantrine in infants and neonates (<5 kg BW)

Impact of CYP3A4 ontogeny profiles on the simulated PK parameters for artemether and lumefantrine in infants and neonates (<5 kg BW)

PK parameter	Population	Observed in B2307 study	CYP3A4 Ontogeny	Predicted	Pred/Obs
**Artemether (5 mg)**					
First dose geometric mean (90% CI) C_{max}, ng/mL	Infants (53-157 days, <5 kg)	68 (45, 103)	Salem	112 (89.7, 140)	1.65
			Upreti	63.5 (49.2, 81.9)	0.93
	Neonates (21-26 days <5 kg)	62.2 (33.6, 115)	Salem	147 (100, 216)	2.36
			Upreti	93.2 (62.4, 139)	1.50
**Lumefantrine (60 mg)**					
6-dose regimen, geometric mean (90% CI) C_{max}, µg/mL	Infants (53-157 days, <5 kg)	3.19 (2.53, 4.00)	Salem	3.78 (3.15, 4.53)	1.18
			Upreti	2.90 (2.42, 3.47)	0.91
	Neonates (21-26 days <5 kg)	4.65 (3.31, 6.53)	Salem	5.64 (3.87, 8.22)	1.21
			Upreti	4.60 (3.21, 6.59)	0.99
6-dose regimen, geometric mean (90% CI) C_{168h}, ng/mL	Infants (53-157 days, <5 kg)	353 (250, 498)	Salem	1102 (934, 1299)	3.12
			Upreti	471 (382, 582)	1.33
	Neonates (21-26 days <5 kg)	615 (403, 937)	Salem	1638 (859, 2766)	2.68
			Upreti	803 (527, 1223)	1.31

BW: body weight; CI: confidence interval; Pred: predicted; Obs: observed

The simulations were conducted using demographics from subjects of infants and old neonates (CALINA)

Salem ontogeny: Salem F, Johnson TN, Abduljalil K, et al. A Re-evaluation and Validation of Ontogeny Functions for Cytochrome P450 1A2 and 3A4 Based on In Vivo Data. Clin Pharmacokinet. 2014; 53(7):625-36.

Upreti ontogeny: Upreti VV, Wahlstrom JL. Meta-analysis of hepatic cytochrome P450 ontogeny to underwrite the prediction of pediatric pharmacokinetics using physiologically based pharmacokinetic modeling. J Clin Pharmacol. 2016; 56(3), 266-83.

Using the Salem ontogeny, the ratios of predicted vs. observed for artemether C_{max}, lumefantrine C_{max} and C_{168h} values CALINA were over-predicted. The use of the Upreti ontogeny improved the PBPK model PK predictions. Therefore, the Upreti ontogeny was used for the final PBPK model to predict artemether and lumefantrine PK parameters in neonates across all age subgroups.
