# Supplementary material for: Physiologically based pharmacokinetic modelling to predict artemether and lumefantrine exposure in neonates weighing less than 5 kg treated with artemether–lumefantrine to supplement the clinical data from the CALINA study
Source: Trop Med Health. 2025 Aug 25;53:116. doi: 10.1186/s41182-025-00790-w (PMC12376358; doi:10.1186/s41182-025-00790-w)

Helen Gu et al. Physiologically-based pharmacokinetic modeling to predict artemether and lumefantrine exposure in neonates weighing less than 5 kg treated with artemether-lumefantrine to supplement the clinical data from the CALINA study

Additional File 4: Model predicted geometric mean values and 90% confidence intervals (90% CI) versus the observed values for artemether C_{max} on Day 1, lumefantrine C_{max} on Day 3, and lumefantrine C_{168h}

Predicted versus observed geometric mean artemether C_{max} in infants and older neonates (BW <5kg and age of >28 days)

Symbols represent individual observed (triangles) and predicted (diamonds) artemether C_{max} values and solid lines, the geometric mean values. Shaded areas are the 90% confidence interval (CI) for observed (white) and predicted (pale gray) data; dark grey shading shows the overlap between the 90% CIs for observed and predicted data. Simulations for the individual patients were conducted using a custom-trial design population in Simcyp according to the exact demographic variables including age, weight, gender, and height from CALINA.

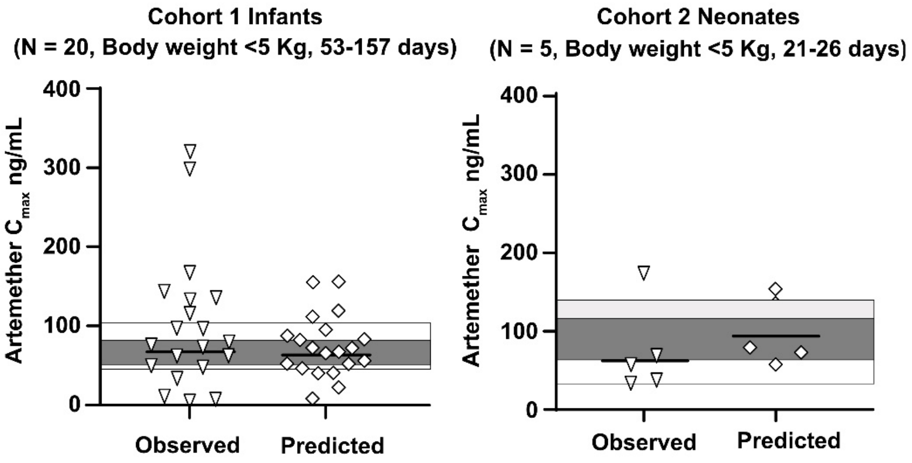

Predicted versus observed geometric mean lumefantrine C_{max} in infants and older neonates (BW <5kg and age of >28 day)

Symbols represent individual observed (triangles) and predicted (diamonds) lumefantrine C_{max} and solid lines geometric mean values. Shaded areas are the 90% CI for observed (white) and predicted (pale gray) data; dark gray shading indicates where 90% CIs for observed and predicted data overlap. Simulations for the individual patients were conducted using a custom-trial design populations in Simcyp according to the exact demographic variables including age, weight, gender, and height from CALINA.

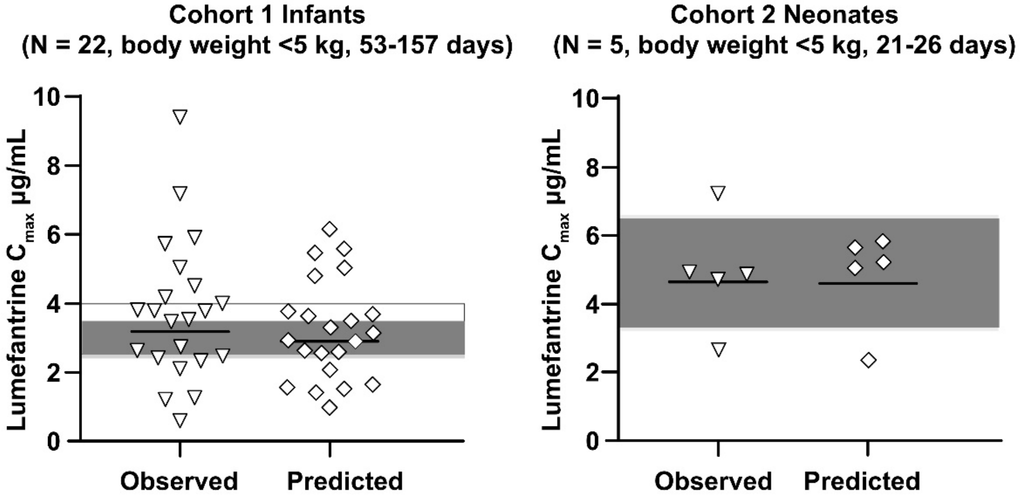

Predicted versus observed geometric mean lumefantrine C_{168h} in infants and older neonates (BW <5kg and age of >28 day)

Symbols represent individual observed (circles) and predicted (squares) lumefantrine C_{168} and solid lines the geometric mean value. Shaded areas are the 90% CI for observed (white) and predicted (pale gray) data. The dark gray areas are the overlaps between 90% CIs for observed and predicted data. Simulations for the individual patients were conducted using a custom-trial design populations in Simcyp according to the exact demographic variables including age, weight, gender, and height from CALINA.

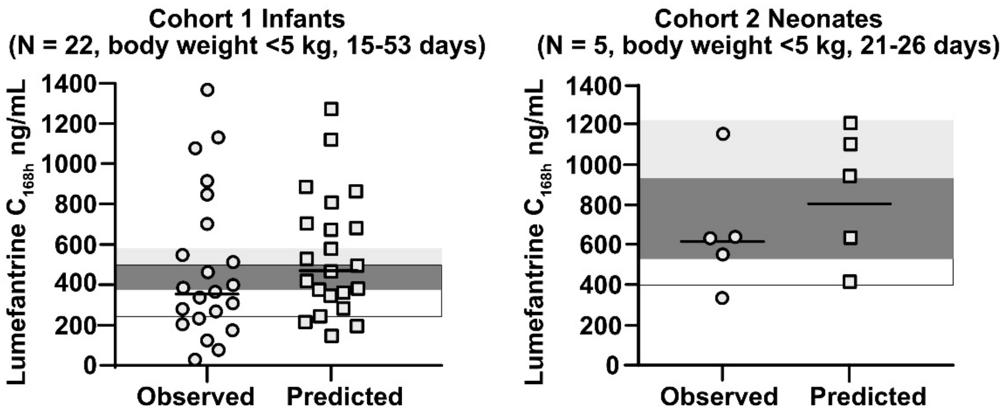

Supplement: Supplementary file 4 — Additional file 4. Model predicted geometric mean values and 90% confidence intervals (90% CI) versus the observed values for artemether Cmax on Day 1, lumefantrine Cmax on Day 3, and lumefantrine C168h. [file 41182_2025_790_MOESM4_ESM.pdf]
