# Supplementary material for: Physiologically based pharmacokinetic modelling to predict artemether and lumefantrine exposure in neonates weighing less than 5 kg treated with artemether–lumefantrine to supplement the clinical data from the CALINA study
Source: Trop Med Health. 2025 Aug 25;53:116. doi: 10.1186/s41182-025-00790-w (PMC12376358; doi:10.1186/s41182-025-00790-w)
Supplement: Supplementary file 5 — Additional file 5. Predicted artemether maximum plasma concentrations (Day 1) in neonates after a first dose of 5 mg artemether + 60 mg lumefantrine. [file 41182_2025_790_MOESM5_ESM.pdf]

Helen Gu et al. Physiologically-based pharmacokinetic modeling to predict artemether and lumefantrine exposure in neonates weighing less than 5 kg treated with artemether-lumefantrine to supplement the clinical data from the CALINA study

Additional File 5: Predicted artemether maximum plasma concentrations (Day 1) in neonates after a first dose of 5 mg artemether + 60 mg lumefantrine

Predicted artemether maximum plasma concentrations (Day 1) in neonates after a first dose of 5 mg artemether + 60 mg lumefantrine

Age range (day)	BW (kg) Mean ± SD	Plasma concentration		
		artemether C_{max}, ng/mL (1st dose on Day 1)		
		Mean ± SD	Median (range) [5th, 95th percentile]	Geometric mean (90% CI)
1 – 28 (n=20)	3.51 ± 0.66	133 ± 72.7	116 (34.4, 307) [46.7, 285]	115 (91.9, 143)
1 – 28 (n=100)	3.53 ± 0.57	126 ± 69.4	111 (29.3, 321) [47.4, 272]	109 (100, 120)
1 – 28 (n=1000)	3.55 ± 0.55	118 ± 60.8	108 (12.0, 366) [39.4, 230]	102 (99.4, 105)
1 – 7 (n=20)	3.17 ± 0.55	182 ± 101	159 (35.2, 418) [65.9, 383]	156 (123, 196)
1 – 7 (n=100)	3.20 ± 0.45	170 ± 93.4	148 (35.2, 418) [57.7, 358]	145 (132, 160)
1 – 7 (n=1000)	3.25 ± 0.45	156 ± 84.8	144 (12.0, 464) [42.4, 311]	131 (127, 135)
8 -14 (n=20)	3.37 ± 0.59	152 ± 90.7	124 (28.2, 366) [51.0, 338]	127 (98.8, 163)
8 -14 (n=100)	3.41 ± 0.49	142 ± 83.5	121 (26.0, 366) [47.8, 315]	120 (108, 132)
8 -14 (n=1000)	3.45 ± 0.49	131 ± 74.2	119 (10.6, 424) [34.6, 266]	109 (106, 113)
15 - 28 (n=20)	3.68 ± 0.65	120 ± 74.9	97.7 (22.4, 300) [39.5, 280]	99.0 (76.3, 128)
15 - 28 (n=100)	3.71 ± 0.55	113 ± 69.9	96.3 (18.9, 308) [39.5, 258]	94.1 (85.0, 104)
15 - 28 (n=1000)	3.76 ± 0.54	105 ± 61.0	95.7 (8.58, 365) [27.5, 222]	87.4 (84.5, 90.4)

BW: body weight; CE: confidence interval; SD: standard deviation

The simulation was conducted in a population across 4 different age ranges of 20 patients/trial (n=20), 20 patients/5 trials (n=100) or 100 patients/10 trials (n=1000). The female ratio was 0.5.
