# Supplementary material for: Physiologically based pharmacokinetic modelling to predict artemether and lumefantrine exposure in neonates weighing less than 5 kg treated with artemether–lumefantrine to supplement the clinical data from the CALINA study
Source: Trop Med Health. 2025 Aug 25;53:116. doi: 10.1186/s41182-025-00790-w (PMC12376358; doi:10.1186/s41182-025-00790-w)
Supplement: Supplementary file 6 — Additional file 6. Model-predicted artemether and lumefantrine Cmax compared with observed data from the CALINA study and Study COA566B2303. [file 41182_2025_790_MOESM6_ESM.pdf]

Helen Gu et al. Physiologically-based pharmacokinetic modelling to predict artemether and lumefantrine exposure in neonates weighing less than 5 kg treated with artemether-lumefantrine to supplement the clinical data from the CALINA study

Additional File 6: Model-predicted artemether and lumefantrine C_{max} compared with observed data from the CALINA study and Study COA566B2303.

Comparison of observed artemether C_{max} in paediatric patients of >5 kg (Study B2303), <5 kg (CALINA study) and predicted in <5kg neonates (PBPK model)

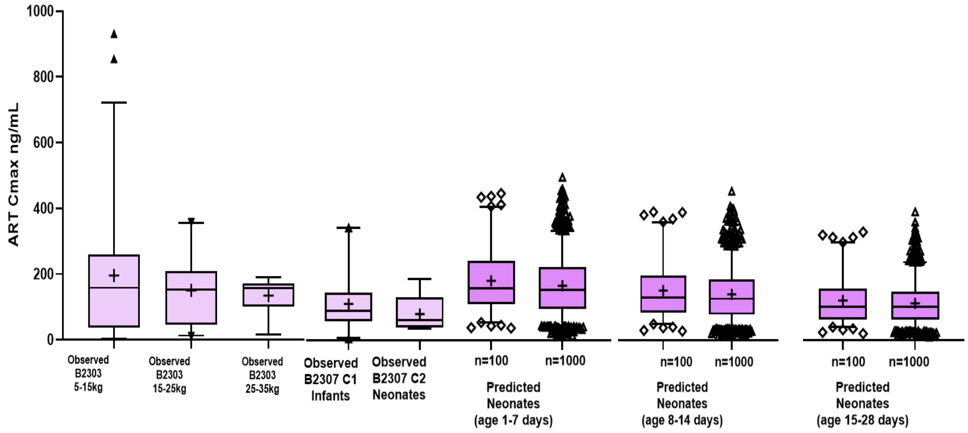

Boxes = 25th to 75th percentiles
Middle line of box = median
Whiskers = 5th to 95th percentiles
+ = mean

Comparison of observed lumefantrine C_{max} in paediatric patients of >5 kg (Study B2303), <5 kg (CALINA study) and predicted in <5kg neonates (PBPK model)

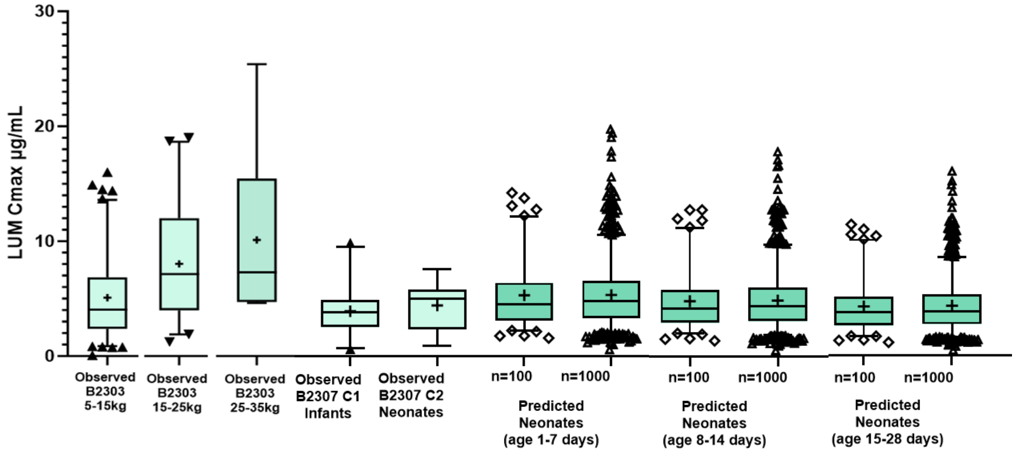

Boxes = 25th to 75th percentiles
Middle line of box = median
Whiskers = 5th to 95th percentiles
+ = mean
