# Supplementary material for: Physiologically based pharmacokinetic modelling to predict artemether and lumefantrine exposure in neonates weighing less than 5 kg treated with artemether–lumefantrine to supplement the clinical data from the CALINA study
Source: Trop Med Health. 2025 Aug 25;53:116. doi: 10.1186/s41182-025-00790-w (PMC12376358; doi:10.1186/s41182-025-00790-w)
Supplement: Supplementary file 7 — Additional file 7. Predicted lumefantrine C168h in neonates for n=20, 100, or 1000 patients. [file 41182_2025_790_MOESM7_ESM.pdf]

Helen Gu et al. Physiologically-based pharmacokinetic modeling to predict artemether and lumefantrine exposure in neonates weighing less than 5 kg treated with artemether-lumefantrine to supplement the clinical data from the CALINA study

Additional File 7: Predicted lumefantrine C_{168h} in neonates for n=20, 100, or 1000 patients

Predicted lumefantrine C_{168h} in neonates after 3-day treatment of 5 mg artemether + 60 mg lumefantrine

	BW (kg) growth range (Day 1 and Day 8) Mean ± SD		lumefantrine C_{168h}, ng/mL		
	Day 1	Day 7	**Mean ± SD**	**Median (range) [5th, 95th percentile]**	**Geometric mean (90% CI)**
1 – 28 (n=20)	3.51 ± 0.66	3.68 ± 0.69	1244 ± 846	1068 (158, 3061) [253, 2910]	959 (703, 1308)
1 – 28 (n=100)	3.53 ± 0.57	3.71 ± 0.59	1215 ± 901	970 (81.7, 4425) [239, 3065]	911 (797, 1042)
1 – 28 (n=1000)	3.55 ± 0.55	3.73 ± 0.57	1242 ± 895	1024 (43.5, 6350) [263, 2997]	968 (925, 1014)
1 – 7 (n=20)	3.17 ± 0.55	3.36 ± 0.59	1657 ± 1035	1514 (283, 4242) [393, 3428]	1336 (1010, 1767)
1 – 7 (n=100)	3.20 ± 0.45	3.40 ± 0.49	1581 ± 1110	1347 (164, 5586) [382, 3944]	1236 (1094, 1397)
1 – 7 (n=1000)	3.25 ± 0.45	3.44 ± 0.48	1567 ± 1039	1332 (90.5, 7816) [394, 3563]	1268 (1215, 1322)
8 -14 (n=20)	3.37 ± 0.59	3.55 ± 0.62	1366 ± 909	1240 (201, 3714) [297, 2884]	1070 (793, 1443)
8 -14 (n=100)	3.41 ± 0.49	3.58 ± 0.51	1312 ± 963	1087 (115, 4740) [301, 3434]	1003 (883, 1140)
8 -14 (n=1000)	3.45 ± 0.49	3.63 ± 0.51	1309 ± 897	1109 (57.3, 6645) [322, 3036]	1043 (998, 1090)
15 – 28 (n=20)	3.68 ± 0.65	3.84 ± 0.67	1067 ± 755	941 (133, 2997) [210, 2365]	808 (586, 1113)
15 – 28 (n=100)	3.71 ± 0.55	3.88 ± 0.57	1039 ± 796	813 (73.4, 3839) [218, 2864]	772 (675, 884)
15 – 28 (n=1000)	3.76 ± 0.54	3.92 ± 0.56	1049 ± 754	858 (34.5, 5813) [226, 2513]	818 (781, 856)

BW: body weight; CE: confidence interval; SD: standard deviation

The simulation with selection of “redefine patients over time” was conducted in a population across 4 different age ranges of 5 patients/4trials (n=20), 5 patients/20 trials (n=100) or100 patients/10 trials (n=1000). The female ratio was 0.5.
