# Supplementary material for: Physiologically based pharmacokinetic modelling to predict artemether and lumefantrine exposure in neonates weighing less than 5 kg treated with artemether–lumefantrine to supplement the clinical data from the CALINA study
Source: Trop Med Health. 2025 Aug 25;53:116. doi: 10.1186/s41182-025-00790-w (PMC12376358; doi:10.1186/s41182-025-00790-w)
Supplement: Supplementary file 8 — Additional file 8. Parameter sensitivity analyses for absorption and clearance input parameters on the predicted plasma concentrations for artemether. [file 41182_2025_790_MOESM8_ESM.pdf]

Helen Gu et al. Physiologically-based pharmacokinetic modeling to predict artemether and lumefantrine exposure in neonates weighing less than 5 kg treated with artemether-lumefantrine to supplement the clinical data from the CALINA study

Additional File 8: Parameter sensitivity analyses for absorption and clearance input parameters on the predicted plasma concentrations for artemether

Artemether C_{max}

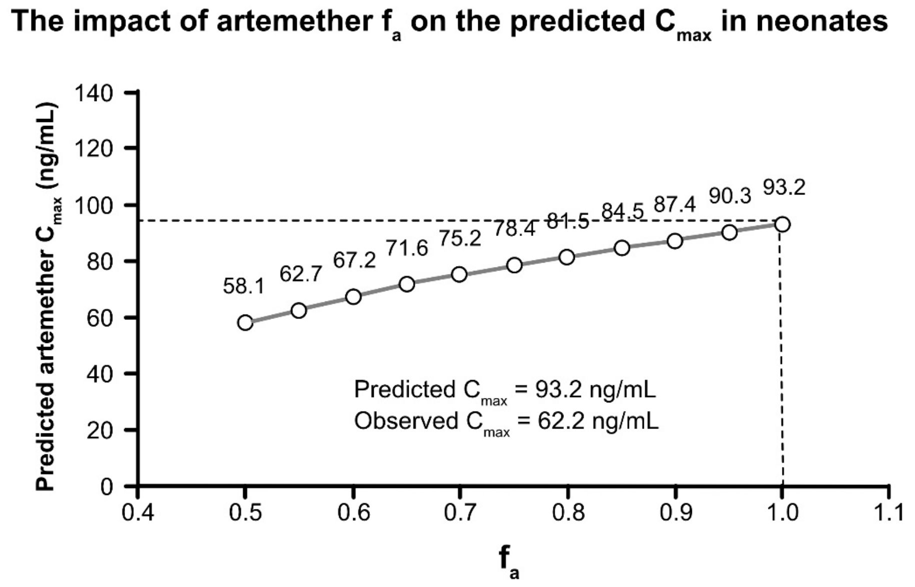

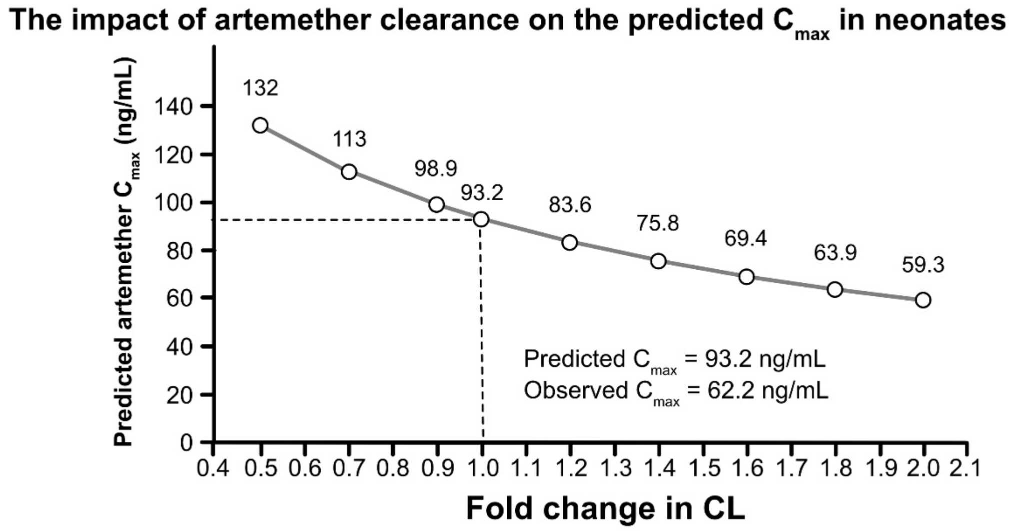

Top: predicted changes in artemether C_{max} when changing the respective model absorption parameters are shown by the gray line with circle symbols. The predicted concentrations using the current model are indicated by the dotted horizontal line. The current parameters used in the model are indicated by the vertical dotted line.

Lower: predicted change in artemether C_{max} with a 2-fold increase in artemether model clearance (CL) and unchanged fm_{CYP3A4} and fm_{CYP2B6} or fm_{CYP3A4} and fm_{add}, is shown as a gray line with circle symbols. The predicted concentrations using the current model are indicated by the dotted horizontal line. The current parameters used in the model are indicated by the vertical dotted line.
