# Supplementary material for: Physiologically based pharmacokinetic modelling to predict artemether and lumefantrine exposure in neonates weighing less than 5 kg treated with artemether–lumefantrine to supplement the clinical data from the CALINA study
Source: Trop Med Health. 2025 Aug 25;53:116. doi: 10.1186/s41182-025-00790-w (PMC12376358; doi:10.1186/s41182-025-00790-w)

Helen Gu et al. Physiologically-based pharmacokinetic modeling to predict artemether and lumefantrine exposure in neonates weighing less than 5 kg treated with artemether-lumefantrine to supplement the clinical data from the CALINA study

Additional File 11: Potential impact of CYP3A7 contributions on newborns (1-7 days, n=100)

Potential impact of CYP3A7 contributions on newborns (1-7 days, n=100). The predicted change in artemether C_{max} and lumefantrine C_{168h} by changing artemether or lumefantrine model parameters is shown as a gray line with circle symbols.

Artemether C_{max}

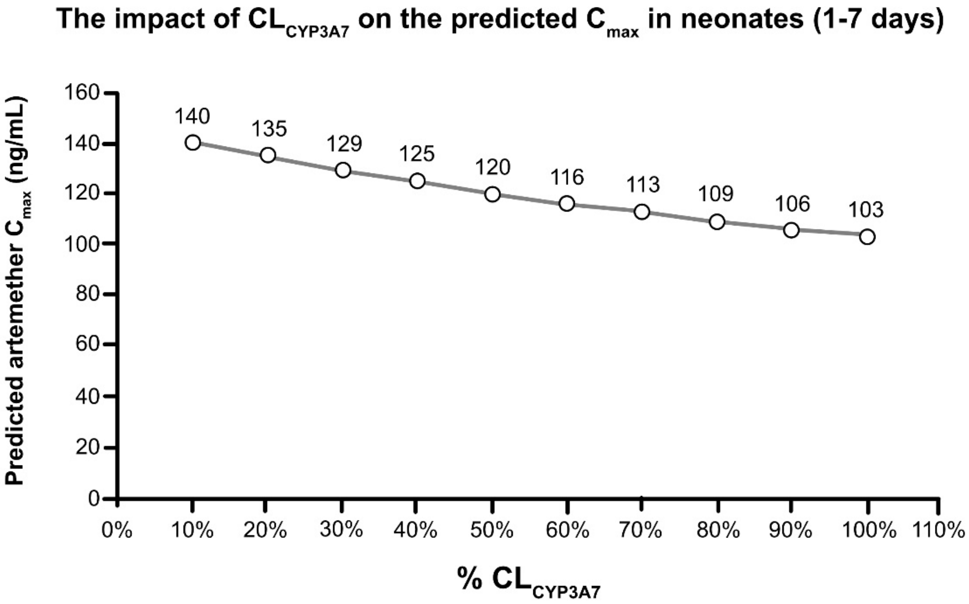

Lumefantrine C_{168h}

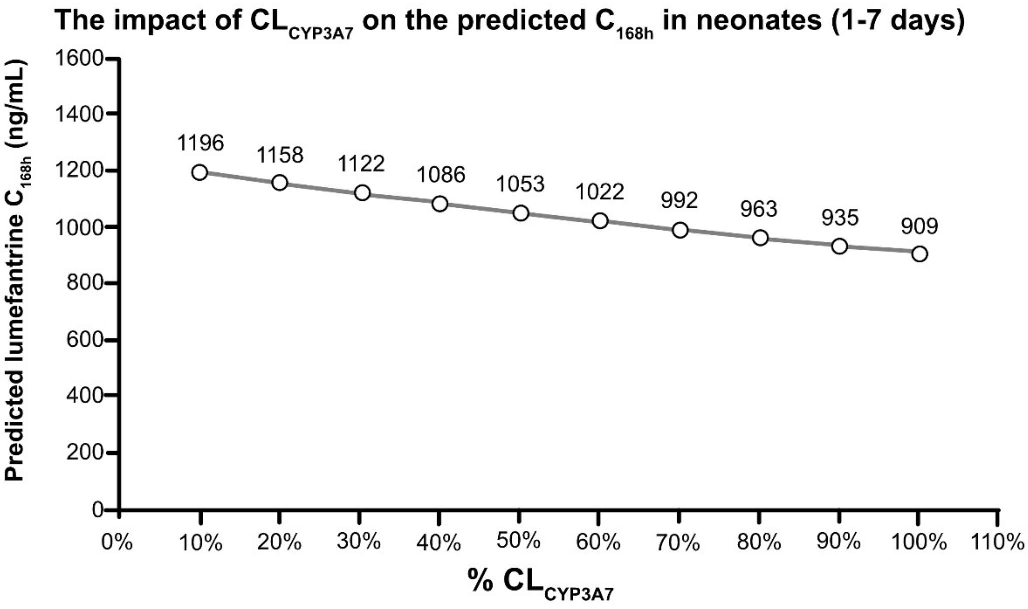

Supplement: Supplementary file 11 — Additional file 11. Parameter sensitivity analysis of CYP3A7 contributions to the overall metabolism on the predicted plasma concentrations in neonates: potential impact of CYP3A7 contributions on newborns (1-7 days, n=100). [file 41182_2025_790_MOESM11_ESM.pdf]
